# Molecular Alterations and Pathways in Intrahepatic Cholangiocarcinoma: Available Evidence and New Perspectives

**DOI:** 10.3390/ijms262411961

**Published:** 2025-12-11

**Authors:** Martina Astore, Laura Fabbri, Andrea Monte, Chiara Deiana, Alessandro Rizzo, Simona Tavolari, Marzia Deserti, Giovanni Brandi, Andrea Palloni, Giorgio Frega

**Affiliations:** 1 Department of Medical and Surgical Sciences (DIMEC), University of Bologna, 40138 Bologna, Italyandrea.monte@studio.unibo.it (A.M.);; 2 S.S.D. C.O.r.O. Bed Management Presa in Carico, TDM, IRCCS Istituto Tumori “Giovanni Paolo II”, Viale Orazio Flacco 65, 70124 Bari, Italy; rizzo.alessandro179@gmail.com; 3 Medical Oncology, IRCCS Azienda Ospedaliero—Universitaria di Bologna, 40138 Bologna, Italy; simona.tavolari@aosp.bo.it; 4 Osteoncologia Sarcomi dell’Osso e dei Tessuti Molli e Terapie Innovative, IRCCS Istituto Ortopedico Rizzoli, 40136 Bologna, Italy

**Keywords:** intrahepatic cholangiocarcinoma, biliary tract cancer, target genes, pathways, oncogene

## Abstract

Intrahepatic cholangiocarcinoma (iCCA) is an aggressive cancer arising within the liver from the bile ducts, and it is characterized by limited therapeutic options and a poor prognosis. This neoplasm exhibits both high intra-tumor and inter-tumor heterogeneity and many oncogenic and tumor suppressor genes are involved in its development and progression. Here, we summarize the major pathways and driver genes involved in the genesis and progression of iCCA, with a special look at their potential therapeutic values. We approach not only the well–known FGFR, IDH and HER2 alterations but also delve into less known cellular pathways such as cell surface receptors, cellular signaling pathways, tumor suppressor genes and metabolic pathways. The aim of our review is therefore not only to summarize the available evidence on singular pathways/alterations but also to foster and promote new investigations into lesser known alterations that could be present in each singular iCCA case.

## 1. Introduction

Biliary tract cancers (BTCs) comprise a heterogeneous group of malignancies affecting the biliary system, including gallbladder cancer (GBC), intrahepatic cholangiocarcinoma (iCCA), extrahepatic cholangiocarcinoma (eCCA), and cancers of the ampulla of Vater [1,2,3]. Together, BTCs account for less than 1% of all malignancies [4]. Among the two main BTCs subtypes, eCCA is more prevalent than iCCA [2,3,4]. While iCCA accounts for about a 10% of all BTCs cases, eCCA represent the vast majority of diagnosis (60–70%) and can be further classified into perihilar cholangiocarcinoma (also known as Klatskin tumor) and distal CCA, with perihilar CCA being the most common subtype [2,3,4,5]. Surgical resection remains the only potentially curative treatment option, but it is feasible in only a small proportion of patients, and recurrence following surgery is frequent. The majority of BTC cases are diagnosed at a locally advanced or metastatic stage, with a median overall survival of around one year. The gold standard first line treatment is the combination of doublet chemotherapy Cisplatin/Gemcitabine and immunotherapy with Durvalumab or Pembrolizumab according to the results of two randomized phase III trials (TOPAZ1 and KEYNOTE-966, respectively) [6,7]. iCCA is characterized by substantial molecular heterogeneity, possibly attributable to the presence of distinct stem cell niches and developmental origins [8,9]. In recent years, new key signaling pathways involved in cholangiocarcinoma pathogenesis and progression have been identified. Furthermore, compared to other malignancies, iCCA exhibits an intermediate mutational rate [10,11]. Recently, next-generation sequencing (NGS) has gained interest due to its broader genomic profiling capabilities and is now recommended as a potential molecular diagnostic tool by the European Society for Medical Oncology (ESMO) and the National Comprehensive Cancer Network (NCCN) guidelines, to identify possible targets for second-line treatment options [12,13]. This review provides an overview of the major driver pathways implicated in iCCA development and progression, beginning with an analysis of key cell surface receptors, followed by a discussion of oncogenic, tumor suppressor, and metabolic pathways involved in the disease. We also reported in Supplementary Table S1 some of the ongoing clinical trials evaluating new potential treatments targeting the molecular alterations here summarized.

## 2. Cell Surface Receptors

### 2.1. HER Family

The Human Epidermal Growth Factor Receptor (HER) family, which comprises four receptors (EGFR/ErbB-1, HER2/ErbB-2, HER3/ErbB-3, and HER4/ErbB-4) [14,15], plays a crucial role in cell proliferation, migration, and invasion. Dysregulation of these receptors and their downstream signaling pathways, including PI3K/AKT/mTOR, Ras/Raf/MAPK, and phospholipase C (PLCγ), has been implicated in tumor progression and it is associated with poor prognosis, although the mechanism by which this happens is still unclear [14]. EGFR overexpression has been reported in 11–30.8% of iCCAs [16]. iCCA tissues exhibit increased levels of phosphorylated EGFR (pEGFR) and phosphorylated STAT3 (pSTAT3) compared with non-cancerous liver tissues [17]. Furthermore, high levels of EGFR, MUC1, MUC4, and fascin expression are associated with poor prognosis [18].

These receptors share a common structural organization, comprising an intracellular component with a tyrosine kinase domain and a C-terminal segment, as well as an extracellular component containing an ErbB ligand-binding domain and a transmembrane lipophilic domain [15]. Typically, the activation of HER family receptors is ligand-dependent, with the ligand binding to the extracellular domain triggering homo- or heterodimerization, phosphorylation of tyrosine residues and subsequent activation of downstream signaling cascades [19,20]. In contrast to the other HER, HER2 activation can also occur independently of epidermal growth factor (EGF) ligand interaction, via spontaneous homodimerization [21]. Once fully activated, HER2 functions as a co-receptor for other HER family members and plays a crucial role in carcinogenesis [22,23,24,25]. The most common HER2 alterations associated with tumor growth and progression include amplification, overexpression, and point mutations. HER2 amplification leads to an increased HER2 gene copy number and corresponding protein expression, whereas HER2 overexpression results in enhanced protein expression on the cell membrane, which is not necessarily due to gene amplification. Additionally, point mutations can induce constitutive activation of the protein, enhancing its oncogenic potential. The most frequently identified HER2 mutations across various cancer types include G660D, V659E, R678Q, and Q709L: these alterations converge to induce hyperactivation of HER2 signaling pathways [26,27]. Among different cancer types, HER2 overexpression and amplification are more prevalent than point mutations, particularly in breast, gastric, colorectal cancers, and non-small cell lung cancer (NSCLC), where specific anti-HER2 targeted therapies have been approved [28,29]. In biliary tract cancers (BTCs), HER2 receptor overexpression has been observed in approximately 15–20% of cases, with a higher frequency in GBC and eCCA [21,30,31]. According to Galdy et al., HER2 gene amplification is detected in up to 30% of BTCs, predominantly in eCCA and GBC, with a rate of 4,8% (95% CI 0–14.5%) in iCCA, whereas point mutations occur in approximately 6.2% of cases, with a higher incidence in eCCA [21,27,32]. Given the low percentage of HER2 altered iCCA, the majority of data available on the subject include all BTC without further subtype distinction. HER2 status is assessed using immunohistochemistry (IHC) to evaluate receptor expression on the cell surface and in situ hybridization (ISH) to confirm gene amplification, with the same criteria of gastric cancer (IHC 0: Negative expression, IHC 1+: Weak expression IHC 2+: Moderate expression (requires ISH confirmation) IHC 3+: Strong expression (HER2-positive) [33,34]. The prognostic significance of HER2 alterations in BTCs remains inconclusive, with conflicting results reported in the literature. Vivaldi et al. demonstrated a statistically significant reduction in progression-free survival (PFS) and a notable, albeit non-statistically significant, reduction in overall survival (OS) in patients with resected BTC harboring HER2 positivity [35]. Similarly, Lowery et al. observed a reduction in time to progression (TTP) during first-line chemotherapy and a shorter OS in patients with NGS-determined HER2-positive advanced or metastatic BTCs [36]. Conversely, a recent trial failed to confirm the prognostic and predictive role of HER2 status in metastatic BTCs treated with cisplatin-based first-line chemotherapy [32]. Given the potential role of HER2 in BTC tumorigenesis, Several clinical trials have explored targeted therapies against this molecular pathway. Notably, the phase IIa basket trial MyPathway, published in 2021, investigated a dual HER2 blockade with trastuzumab and pertuzumab in pretreated HER2-amplified and/or overexpressed BTCs. The study reported an objective response rate (ORR) of 23%, mPFS of 4.0 months, and mOS of 10.9 months, with an acceptable safety and tolerability profile [34]. Encouraged by these findings, the humanized bispecific monoclonal antibody (mAb) zanidatamab was evaluated in the HERIZON-BTC-01 study, which enrolled pretreated patients with advanced HER2-amplified BTCs, showing promising survival and response rate outcomes [37]. Additional treatment strategies, including mAb and tyrosine kinase inhibitors (TKIs) such as trastuzumab or tucatinib, as well as combinations of mAb with chemotherapy, have demonstrated promising efficacy [37,38,39,40,41]. Another emerging therapeutic approach involves antibody–drug conjugates (ADCs), particularly trastuzumab deruxtecan (DXd), which was assessed in the DESTINY-PanTumor02 trial [42]. Currently, DXd is FDA-approved as a tumor-agnostic therapy for HER2 3+ advanced BTCs that have progressed following standard first-line treatment.

### 2.2. FGF Receptors

The Fibroblast Growth Factor (FGF) signaling pathway is involved in various biological functions including cell survival, proliferation, differentiation, wound repair, migration, and angiogenesis [43].

The mammalian FGF superfamily includes four transmembrane tyrosine kinase receptors (RTKs), FGFR1-4, and 18 FGF ligands [44].

Upon release, FGF ligands bind to monomeric receptors, inducing dimerization and autophosphorylation of the intracellular domain [43]. This activation triggers several intracellular signaling cascades, including the RAS-dependent mitogen-activated protein kinase (MAPK), phosphatidylinositol 3-kinase (PI3K)/Akt/mTOR, phospholipase Cγ (PLCγ), and JAK/STAT pathways [45].

Given these physiological roles, molecular aberrations in FGFR signaling—such as mutations, insertions, deletions, gene fusions, and translocations—can significantly contribute to cancer pathogenesis. These aberrations have been detected in patients with intrahepatic cholangiocarcinoma (iCCA).

The most frequently observed alteration in iCCA is FGFR2 fusion, with a prevalence ranging from approximately 10% to 16%, and it is typically mutually exclusive with KRAS/BRAF mutations [46]. FGFR2 fusion-positive iCCA represents a distinct molecular subtype of biliary tract cancer. This rearrangement is more prevalent in females and younger age patients. Furthermore, it seems to be associated with longer OS compared to wild-type phenotype [47].

The introduction of FGFR inhibitors, such as pemigatinib and futibatinib, has represented an important step forward in the treatment of iCCA with FGFR-2 alterations [48,49]. Furthermore, the development of secondary mutations in the FGFR2 kinase domain has been identified as a major mechanism of tumor progression and treatment resistance in FGFR2 fusion-positive patients treated with targeted inhibitors [31].

### 2.3. PDGF Receptors

The Platelet-Derived Growth Factor (PDGF) and its receptors (PDGFRs) play a pivotal role in various biological processes, including mesenchymal cell activation, tissue repair, and wound healing. Overexpression of PDGF/PDGFR has been observed in numerous epithelial cancers, including BTC, and is generally associated with poor prognosis [50,51]. These mutations are central to the “proliferation” molecular subclass, as opposed to the “inflammation” class, in the classification proposed by Sia et al. [52]. Hypoxia has been shown to induce overexpression of PDGF-DD in CCA cells. Notably, PDGFR-β immunoreactivity is reported in approximately 90% of iCCA and 70% of eCCA samples [53]. Myofibroblast-derived PDGF-BB has been found to confer cyto-protection to CCA cells by preventing TRAIL-induced cytotoxicity through a Hedgehog signaling-dependent mechanism. Inhibition of PDGFR-β signaling using imatinib mesylate has been shown to promote TRAIL-induced apoptosis in CCA cells, both in vitro and in vivo [54].

### 2.4. VEGF Receptors

Vascular endothelial-derived growth factor (VEGF) family consist of a group of proteins including VEGF-A, VEGF-B, VEGF-C, VEGF-D, VEGF-E and placental growth factor (PGF) [55]. These factors interact with distinct tirosine kinase trans-membrane receptors including VEGFR1-2-3 [55,56]. VEGF plays a pivotal role in the pathogenesis of iCCA by promoting angiogenesis, lymphangiogenesis, and tumor progression. The overexpression of VEGF, particularly VEGF-A and VEGF-C, in iCCA tissues is linked to increased microvascular and lymphatic density, which supports tumor growth, invasion, and early metastatic spread [57,58]. VEGF also promotes vasodilation and enhances vascular permeability [59]. Additionally, VEGF influences lymphatic endothelial cells, cancer-associated fibroblasts, and indirectly affects immune cells within the iCCA microenvironment, driving lymphangiogenesis, vascular remodeling, and metastatic dissemination. Overall overexpression of VEGF protein has been reported in around 50% of iCCA cases [60,61]. Clinically, high VEGF expression correlates with adverse outcomes, including reduced overall survival (HR = 1.93 95% CI 1.52–2.46)., higher rates of lymph node metastasis, and advanced TNM stage [61]. The tumor microenvironment, particularly cancer-associated fibroblasts, can be stimulated by factors such as PDGF-D to secrete VEGF-A and VEGF-C, amplifying lymphangiogenesis and facilitating tumor cell intravasation via VEGFR2 and VEGFR3 expressed on lymphatic endothelial cells [54]. Dual inhibition of FGFR and VEGFR pathways has demonstrated synergistic suppression of lymph-angiogenesis and tumor progression in preclinical models, highlighting the therapeutic potential of targeting VEGF/VEGFR signaling in iCCA [53]. VEGF signaling activates downstream pathways such as PI3K/Akt/mTOR and HIF-1α, which drive endothelial cell proliferation, migration, and survival, as well as upregulation of glycolytic enzymes like hexokinase 2, further supporting tumor metabolism and immune evasion [53].

Collectively, these findings underline the importance of the VEGF/VEGFR axis in iCCA biology and its relevance as a prognostic biomarker and its potential therapeutic target. VEGF/VEGFR-targeted therapies have not demonstrated clinically meaningful efficacy as monotherapy and are not currently standard of care for this indication: early-phase trials of VEGF pathway inhibitors, such as sorafenib, sunitinib, and regorafenib, have shown limited activity in iCCA, with low objective response rates and modest progression-free survival, and are not recommended as single agents [62,63,64]. Ongoing studies are evaluating VEGF/VEGFR inhibitors in combination with chemotherapy and immunotherapy, aiming to exploit synergistic effects and overcome resistance mechanisms [65,66]. Inhibition of VEGF signaling in CCA seems to synergize with immune checkpoint blockade by inducing a pro-inflammatory B cell response [67].

### 2.5. IGF Receptors

Another pathway that is implicated in the pathogenesis and progression of CCA is the insulin-like growth factor 2 (IGF2) signaling axis: its role extends beyond tumor growth, influencing metastasis, immune evasion, and therapy resistance. IGF2 is overexpressed in tumor cells and myofibroblasts, where it activates the insulin receptor (IR) and IGF1 receptor (IGF1R), promoting cellular survival and proliferation [68]. These findings highlight the potential of targeting the IGF2/IGF1R axis as a therapeutic strategy to overcome drug resistance in BTC. IGF2 has a dual role in promoting tumor growth while contributing to metastatic dissemination and immune evasion: it seems that this pathway enhances the epithelial–mesenchymal transition (EMT), a critical process of cancer metastasization, by activating downstream signaling pathways such as MAPK and PI3K/Akt. Moreover, IGF2-mediated suppression of immune cell activity contributes to tumor immune escape [69]. In addition, IGF2 overexpression in CCA seems to be implicated in EGFR-TKI treatment resistance, however there are no approved drugs targeting this receptor thus far [69].

### 2.6. TGF Receptors

The TGF-beta pathway comprises two types of transmembrane receptors, type I and type II TGF receptors (TbetaRI and TbetaRII) and extracellular ligands whose reciprocal binding leads to a heterotetrameric structure composed by TbetaRII and TbetaRI, and in the subsequent phosphorylation of TbetaRI by TbetaRII. The phosphorylation of TbetaRI determines the activation of its serine–threonine kinase intracellular domain that, once activated, generates a stepwise phosphorylation of some proteins of SMAD family, in particular SMAD2 and SMAD3. These proteins both interact with a third SMAD member, SMAD4, a common message transducer. The SMAD complex then translocates in the nucleus through importin-1 and CAN/Nup214 and interacts with different gene promoters finally regulating the transcription process [70,71,72]. TGF-beta receptors can also activate other downstream effectors like MAP kinase (MAPK), phosphatidyl-inositol-3 kinase (PI3K) and have a crosstalk with other signaling routes involved in CCA pathogenesis, such as WNT, HIPPO, NF-B, Notch, hedgehog, JAK/STAT, MAPK, and PI3K-AKT [73,74]. TGF-beta’s pathway in condition of physiological activation is involved in the process of epithelial to mesenchymal transition, in the cell cycle arrest, induction of apoptosis and epigenetic regulation [75,76,77]. Many different alterations of TGF-beta’s pathway have been found in BTC cells. SMAD4 inactivation due to chromosomal deletion or gene mutations has been reported in 15–50% of iCCAs [78,79]. The loss of SMAD4 expression has been associated with negative prognostic factors such as lymph node and intrahepatic metastasis, high histological grade and advanced TNM stage [80]. TGF-beta has been evaluated as a potential therapeutic target in CCA mainly in preclinical models. Currently, a phase II clinical trial is evaluating the activity of a bifunctional antibody targeting PD-L1 and TGF-beta in locally advanced or metastatic second line biliary tract cancer [81].

### 2.7. Notch Receptors

The NOTCH pathway regulates various cellular processes, including differentiation, stemness, embryonic development, angiogenesis, and cell cycle progression, and is implicated in BTC carcinogenesis [82,83]. It comprises four transmembrane receptor proteins, Notch 1, 2, 3, and 4, which form heterodimers, and five ligands: Jagged-1 (JAG-1), JAG-2, Delta-like ligand 1 (Dll1), Delta-like ligand 3 (Dll3), and Delta-like ligand 4 (Dll4).

Intercellular contact activates the Notch signaling pathway, leading to cleavage of the Notch receptor from the membrane by ADAM metallopeptidase domain 10 and 17 (ADAM10 and ADAM17) proteases. The Notch intracellular domain (NICD) then translocates to the nucleus, where it binds the Recombination Signal Binding Protein for Immunoglobulin Kappa J (RBP-J) transcription factor. Upon removal of co-repressors and recruitment of the coactivator Mastermind-like proteins (MAML-1, 2, and 3), downstream mediators are activated, exerting regulatory effects on transcription and translation. The array of effector genes regulated by the Notch pathway is extensive and includes transcriptional inhibitors, oncogenes, tumor suppressors, and cell cycle regulators [84,85]. The best-characterized Notch gene targets belong to the hairy/enhancer of split (HES) and hairy/enhancer of split-related YRPW motif (Hey) families [83].

The Notch pathway plays a critical role in the morphogenesis and remodelling of the biliary tract, cholangiocyte differentiation, and metabolic homeostasis, particularly in lipid and glucose production and storage [86]. In BTC cells, Notch signalling is often hyperactivated, which is associated with poor prognosis and elevated levels of circulating oncogenic markers [87]. The complexity of the Notch cascade is further highlighted by its extensive crosstalk with other key BTC-associated pathways, such as Hippo, AKT/mTOR, VEGF, TGFβ, Hedgehog, EGFR, and Wnt/β-catenin [88].

Given its important role in cancer progression, the Notch pathway has emerged as an attractive therapeutic target. The first class of Notch-targeting agents identified was Gamma-Secretase Inhibitors (GSIs), which prevent cleavage of the receptor’s intracellular domain, thereby blocking downstream signalling. These compounds have been evaluated in various cancer types, although only one phase I trial has assessed their therapeutic implications in CCA patients [89,90]. Additionally, several similar pharmacological agents are currently under investigation in preclinical and clinical studies [91,92,93].

Other therapeutic approaches targeting Notch alterations include anti-Notch antibodies, RNA-interfering antisense oligonucleotides, anti-microRNA therapies, stapled peptides, small-molecule inhibitors (SMIs), and the Inhibitor of Mastermind Recruitment-1 [89,94,95,96,97].

### 2.8. HGF/c-MET

The MET proto-oncogene encodes the receptor tyrosine kinase c-MET, which serves as the receptor for hepatocyte growth factor (HGF). Upon HGF binding, c-MET undergoes dimerization and autophosphorylation, leading to the activation of multiple downstream signaling pathways, including MAPK, PI3K/AKT, SRC, and JAK/STAT. Physiologically, the HGF/c-MET axis is integral to various cellular processes such as survival, proliferation, differentiation, motility, angiogenesis, and tissue regeneration [98]. Deregulation of the HGF/c-MET pathway can result from MET overexpression, genomic amplification, mutations, or alternative splicing. Such aberrations have been implicated in the pathogenesis of several malignancies, including iCCA [99]. In iCCA, c-MET overexpression has been observed in approximately 21.4% of cases and its presence is associated with advanced disease stages and larger tumor volumes, suggesting its role as a negative prognostic factor [16]. Additionally, c-MET overexpression has been linked to the presence of hepatolithiasis and older age [100]. Notably, there is significant crosstalk between the MET, VEGFR, and EGFR pathways, which may contribute to reduced response duration to targeted therapies. For instance, MET amplification has been associated with resistance to anti-EGFR and anti-ERBB2 inhibitors [98]. The multifaceted role of the HGF/c-MET axis in oncogenesis has spurred the development of targeted therapies.

### 2.9. ALK/ROS1 Receptors

Anaplastic lymphoma kinase (ALK) is a receptor tyrosine kinase (RTK) primarily expressed in the central nervous system, small intestine, and testis, where it plays a significant role in various cellular processes, including proliferation, migration, survival, and angiogenesis [101]. Activation of ALK occurs through the binding of secreted growth factors such as pleiotrophin and midkine, leading to the stimulation of downstream signaling pathways, notably RAS/RAF/MEK/ERK, PI3K/AKT, and JAK/STAT [101]. In iCCA, ALK alterations are exceedingly rare. A sequencing study reported one case of EML4-ALK fusion among the 109 iCCAs included [79]. Currently, the efficacy of ALK inhibitors in treating iCCA is under investigation in phase II basket clinical trials.

The ROS1 oncogene encodes a transmembrane RTK that shares approximately 49% amino acid sequence identity with ALK, resulting in similar physiological functions. This homology also means that inhibitors targeting ALK often exhibit activity against ROS1. However, in iCCA, ROS1 rearrangements are similarly uncommon. For example, a study involving 208 iCCA and 53 eCCA cases identified ROS1 rearrangements in only three iCCA cases (1.4%) [102]. The low incidence of ROS1 rearrangements in iCCA suggests that such genetic alterations are rare and may be influenced by ethnic and environmental factors [103,104]. Consequently, while ALK and ROS1 inhibitors have shown promise in other cancers, their applicability in iCCA remains limited due to the rarity of these genetic alterations.

### 2.10. TRK Receptors

The tropomyosin receptor kinase (TRK) receptor family comprises three transmembrane receptor called TRK A, B, and C (TRKA, TRKB, and TRKC) encoded by the correspondent NTRK1, NTRK2, and NTRK3 genes [105,106]. These receptors are extremely important in nervous system development during embryogenesis, but after this evolutive period, their expression is limited to the nervous system, testis, and smooth muscle. Neurotrophic tropomyosin receptor kinase (NTRK) fusions have been detected in about 1% of all adult solid neoplasia, with an estimated prevalence of 0.25–0.67% in BTCs [107]. According to available data, the prevalence of NTRK gene fusions in iCCA is approximately 0.25% [108]. In NTRK rearranged tumors there are several possible fusion partners, among which the most common are ETV6 and BCAN, leading to hyperactivation of the signalling pathway and stimulation of downstream signaling pathways, including MAPK, PI3K, and PKC with the effect of promoting neuron growth, differentiation, and survival [109]. In everyday clinical practice the detection of NTRK gene fusions can be obtained through different molecular techniques, including IHC, NGS, and liquid biopsy. The interest in this pathway has gradually increased since the identification of two potent inhibitors, larotrectinib and entrectinib, with a wide spectrum of action and efficacy not only in NTRK altered cancer cells but also in cases where other altered genes are present, such as ALK or ROS1. Nowadays, larotrectinib and entrectinib are FDA-approved agnostic drugs in the treatment of NTRK rearranged solid neoplasia [110,111].

### 2.11. RET Receptor

RET (REarranged during Transfection) alterations are considered oncogenic drivers in different type of cancers such as lung and thyroid cancer. Although these tumour types account collectively for the majority of RET fusion-positive cancers, RET fusions have also been observed in several other tumour types at a frequency of less than 1%, including cancers of the breast, colon, esophagus, ovary, prostate, stomach, pancreas, salivary gland, connective tissues, and iCCA. RET fusions lead to a constitutively active, ligand-independent activation of the RET pathway. Selpercatinib, a highly selective RET kinase inhibitor with CNS activity, was developed specifically to treat patients with RET-altered cancers. It showed a notable efficacy in both treatment-naive and pretreated populations in a phase 1/2 study in patients with RET-altered advanced solid tumours [112]. These data led to the global regulatory approval of selpercatinib for RET fusion- positive lung and thyroid cancers. Concurrently this study enrolled a tumour-agnostic population of patients with RET fusion-positive advanced solid tumours.

**Figure 1:**
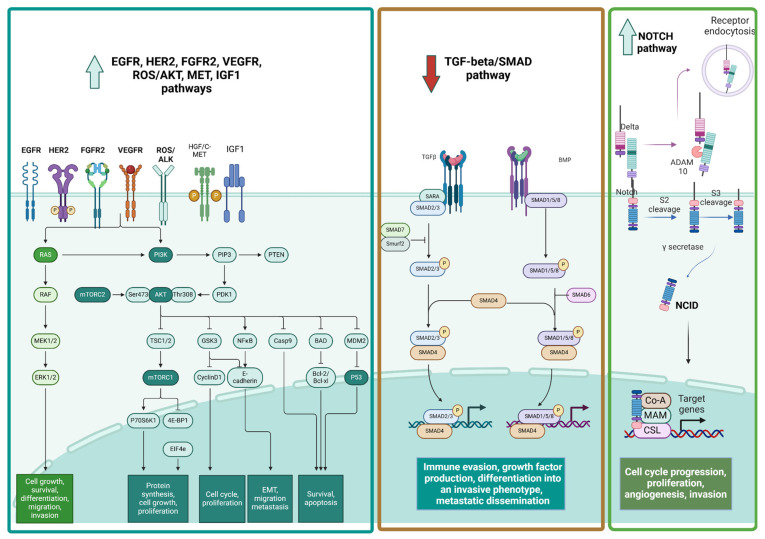
Cell surface receptors and related signaling pathways. Upward arrows indicate upregulation and downward arrows indicate downregulation of target protein.

We summarized cell surface receptors and related signaling pathways in Figure 1.

## 3. Cellular Signaling Pathways

### 3.1. KRAS/BRAF/MEK/ERK Pathway

The RAS-RAF-MEK-ERK signalling cascade, also known as the MAPK/ERK pathway, is a key regulator of cell proliferation, growth, senescence, and survival [113]. The Ras (Rat Sarcoma Virus) proto-oncogene family comprises N-RAS (chromosome 1), H-RAS (chromosome 11), and K-RAS (chromosome 12). Upon activation by extracellular ligands (e.g., EGF, PDGF), RAS transitions from an inactive GDP-bound state to an active GTP-bound state, recruiting RAF-1 kinase to the plasma membrane and triggering RAF activation [114]. BRAF, the most potent MEK activator, subsequently phosphorylates MEK1 and MEK2, which then activate ERK1 and ERK2. These phosphorylated kinases dimerize and translocate to the nucleus, where they regulate essential cellular functions [115].

Mutations in K-RAS occur in 15–25% of iCCAs [116,117]. A specific irreversible inhibitor of the KRAS mutation G12C, sotorasib, is currently being investigated for therapeutic efficacy in this setting, given its use in different cancers with the same mutations such as non-small cell lung cancer.

The BRAF proto-oncogene encodes a serine–threonine protein kinase involved in cell growth and proliferation. BRAF mutations result in constitutive pathway activation, promoting tumorigenesis in multiple malignancies, including BTC. The prevalence of BRAF mutations in iCCA varies between 1% and 22% across different studies [118]. BRAF mutations are linked to worse overall survival (OS) and chemotherapy resistance [117]. In a phase II basket trial, vemurafenib, a BRAF inhibitor, was evaluated in BRAF-mutated iCCA, with only one of eight patients achieving a durable partial response [119]. The phase II ROAR study demonstrated encouraging results for the combination of dabrafenib and trametinib in this population [120].

Additionally, a phase I/II study is investigating DCC-3116, a TKI MET inhibitor, both as monotherapy and in combination with RAS/MAPK pathway inhibitors, in patients with advanced or metastatic solid tumors harboring RAS/MAPK pathway mutations (NCT04892017) [121].

### 3.2. PI3K/AKT/mTOR Pathway

The phosphatidylinositol 3-kinase (PI3K)/AKT/mTOR signaling pathway plays a crucial role in various molecular processes, including cellular metabolism, cell cycle regulation, cell motility and metastasis, transcription, and angiogenesis. The PI3K cascade is activated by various endogenous stimuli and it is triggered primarily by receptor tyrosine kinases (RTKs), including insulin-like growth factor receptor 1 (IGFR1), VEGFR, and EGFR, as well as G-protein-coupled receptors (GPCRs) and RAS family oncogenic proteins [122].

Upon activation, PI3K catalyzes the conversion of phosphatidylinositol-4,5-bisphosphate (PIP2) into phosphatidylinositol-3,4,5-trisphosphate (PIP3), which serves as a docking site for phosphoinositide-dependent kinases (PDK1/2). This interaction leads to the downstream activation of AKT, a serine/threonine kinase, followed by the phosphorylation of tuberous sclerosis complex (TSC1/2), ultimately leading to the activation of the mTOR pathway [123]. The activation of PI3K is primarily regulated by growth factor receptor protein tyrosine kinases, whereas PTEN (phosphatase and tensin homolog) serves as a negative regulator of the PI3K/AKT signaling pathway. PTEN catalyzes the dephosphorylation of PIP3 back to PIP2, thereby inhibiting pathway activation [124].

mTOR functions as a core component of two distinct protein complexes: mTORC1 and mTORC2, each of which participates in different cellular functions. mTORC1 facilitates the dissociation of eukaryotic initiation factor 4E (eIF4E)-binding protein 1 (p-4E-BP1) from eIF4E, thereby enabling eIF4E-mediated regulation of protein synthesis. Additionally, it activates S6 kinase 1 (S6K1, also known as p70S6K), which modulates mRNA translation, and it plays a role in steroidogenesis through the regulation of sterol regulatory element-binding proteins (SREBP1/2) and peroxisome proliferator-activated receptor gamma (PPARγ). Furthermore, mTORC1 is involved in hypoxia signaling, epithelial-to-mesenchymal transition (EMT), and vascular proliferation. In contrast, while the functions of mTORC1 are well characterized, the precise role of mTORC2 remains incompletely understood [125].

The role of the PI3K/AKT/mTOR pathway in BTC carcinogenesis has been extensively investigated in both in vitro and in vivo studies. The most common PI3K alterations observed in BTC specimens involve multiple components of this signaling pathway, including activating mutations or aberrant phosphorylation with overexpression of PIK3CA and PIK3R1, genes encoding different PI3K subunits, AKT mutations, and inactivating mutations of PTEN, among others. Additionally, pathway overactivation can result from alterations in membrane receptors and their ligands, including growth factors, inflammatory cytokines, pro-angiogenic molecules, and stromal-derived peptides [126,127].

In BTC, constitutive activation of the PI3K/AKT pathway is primarily attributed to PTEN inactivation. PTEN suppression, either through loss of expression or phosphorylation-mediated inactivation, has been reported in numerous cases [128]. Gain-of-function mutations in PI3K have been identified in approximately one-third of CCA cases, with activating mutations typically localized in the helical and kinase domains and associated with poor prognosis [129,130,131,132]. On the other hand, epigenetic alterations of PIK3CA are relatively rare, accounting for 0–9% of all genetic mutations. PIK3CA amplification has been detected in 6% of BTC, and it has been correlated to sarcomatoid dedifferentiation [133].

mTOR amplification and overexpression have been reported in 48–92% of BTC cases and are generally associated with moderate differentiation and localized disease, although its prognostic significance remains controversial. The wide range of its reported incidence can be attributed to several factors, such as differences in inclusion criteria, methodological variations for identification of such alterations and differences in study size [134,135]. Additional alterations in CCA development include overexpression of mTOR downstream effectors such as eIF4E, p-4E-BP1, and p70S6K, observed in 22–84% of cases. Furthermore, PTEN loss, resulting from inactivating mutations, transcriptional silencing, or translational impairment, has been identified in 4–71% of BTC cases, with such a wide range possibly reflecting a different biology according to the primary site of the BTC [136,137,138]. The relationship between the PI3K pathway and CCA is closely linked to chronic inflammation, a major risk factor for biliary tract carcinogenesis. Pro-inflammatory cytokines such as interleukin (IL)-6, IL-33, leukemia inhibitory factor (LIF), CCL5, stromal cell-derived factor-1 (SDF-1), and prostaglandins can activate PI3K signaling, promoting cell proliferation, invasion, and resistance to chemotherapeutic agents [139,140,141]. IL-6 and LIF specifically exert anti-apoptotic effects by upregulating the tumor suppressor myeloid cell leukemia-1 (Mcl-1), while IL-33 facilitates tumor cell invasion and metastasis [142]. Additionally, chronic parasitic hepatic infections, such as those caused by Opisthorchis viverrini and Clonorchis sinensis, promote CCA development by releasing factors such as glutathione S-transferase, granulin, and taurocholate, which in turn hyperactivate the PI3K pathway, thereby stimulating cell proliferation and division [143,144,145]. Given its central role in CCA pathogenesis, the PI3K/AKT/mTOR pathway has emerged as a promising therapeutic target, with several clinical trials investigating targeted agents at various levels of the signaling cascade. The most extensively studied drug classes include mTOR inhibitors, PI3K inhibitors, and AKT inhibitors.

### 3.3. JAK/STAT Pathway

The Janus tyrosine kinase-signal transducer and activator of transcription (JAK-STAT) pathway regulates various cellular mechanisms and functions, including cell proliferation, differentiation, survival, migration, and death. Multiple hormones (such as prolactin, growth hormone, leptin, and erythropoietin), cytokines (such as IL-6), and growth factors can activate this signaling cascade by interacting with their respective receptors, leading to the activation of JAKs [146]. This, in turn, triggers the recruitment, phosphorylation, dimerization, and nuclear translocation of STAT proteins, where they modulate transcription by interacting with the promoter regions of a wide range of genes. In doing so, STATs exert an inhibitory effect on other signaling pathways, such as NF-κB, AP-1, and the glucocorticoid receptor pathway, due to the close proximity of their respective binding sequences [147]. This pathway maintains internal balance through a negative feedback mechanism: STAT proteins have recognition sites on the promoter regions of genes encoding suppressors of cytokine signaling 1 (SOCS1) and SOCS3. These suppressors are upregulated, thereby inhibiting STAT activation. While SOCS1 and SOCS3 are the most well-known STAT suppressors, other inhibitors, such as protein inhibitors of activated STAT proteins (PIAS), have also been identified [148]. Different types of JAKs and STATs have specific regulatory functions. Among them, STAT3 and STAT5 are the most expressed proteins in various tissues, playing a key role in maintaining homeostasis and signal transmission. In contrast, STAT1, STAT2, STAT4, and STAT6 have a more specialized distribution and they are primarily involved in immune defence [149]. Alterations in the JAK/STAT pathway with overexpression of different cascade components have been linked to tumorigenesis in both solid and hematologic malignancies, with mutations in JAK1, JAK2, STAT3, and STAT5 being the most frequently observed [150]. Several factors can contribute to BTC development by stimulating the JAK-STAT cascade, with prolactin, leptin, erythropoietin, growth hormone (GH), and IL-6 being the most well-characterized in the literature [151].

Chronic inflammation driven by IL-6 is a recognized risk factor for iCCA. Nearly 50% of iCCA cases, and potentially up to 70% of the inflammation-associated iCCA subtype, exhibit activation of this pathway. IL-6, released during inflammation, acts as an autocrine or paracrine signal, triggering dimerization of its receptor (gp130) and subsequent activation of the JAK/STAT3 signaling pathway. STAT3 can also be further activated by EGFR, ultimately promoting cell proliferation. This leads to the activation of genes primarily involved in cell growth, proliferation, resistance to apoptosis, and angiogenesis [152]. Huang and colleagues recently demonstrated the efficacy of a JAK inhibitor aruxolitinib, either alone or in combination with Gemcitabine, in reducing tumor growth in a CCA cell line. Furthermore, ruxolitinib has been demonstrated to reduce the expression of ALDH1A3 via the JAK/STAT1/3 pathway in CCA cells. Elevated aldehyde dehydrogenase (ALDH) activity correlates with greater drug resistance and metastatic potential across multiple cancer types and the ALDH superfamily—particularly ALDH1A3—plays a key role in driving malignant progression and gemcitabine resistance in patients with advanced CCA [153]. The cytotoxic effect observed warrants further investigation in human trials [154].

### 3.4. Phospholipase C (PLCγ) Pathway

The phospholipase C gamma (PLCγ) pathway plays a pivotal role in tumor initiation, progression, and response to treatment, particularly through its interaction with other pathways. When activated, receptors such as FGFR or growth factors such insulin-like growth factor 1 (IGF-1), can promote phosphorylation of nuclear PLC-β1 while activating nuclear ERK, thereby initiating the inositol phosphate cycle [155]. Moreover, miRNA-205 can activate subsets of liver cancer stem cells and sustain their stem-cell properties by modulating the upstream target PLC-β1 [156]. This activation triggers the production of inositol trisphosphate (IP3) and diacylglycerol (DAG), which in turn activate downstream signaling pathways such as Protein Kinase C (PKC) and promote the release of intracellular calcium (Ca2+). These signaling events contribute to key cellular processes including tumor cell proliferation, survival, and migration [157]. Furthermore, in iCCA, the activation of PLCγ by FGFR signaling induces changes in the tumor microenvironment that promote metastasis: PLCγ activation enhances the expression of key EMT markers, such as N-cadherin and vimentin, while suppressing epithelial markers like E-cadherin. This shift in cellular phenotype facilitates the invasive potential of CCA cells, promoting their ability to disseminate and form secondary tumors [158].

### 3.5. HIPPO/YAP1 Pathway

Overexpression of YAP1 pathway has been found in 32% of iCCA specimen in a study by Sugimachi et al., and its presence was also shown to correlate with poor outcome. This data, along with other similar evidence, supports a potential role of this pathway in iCCA pathogenesis [159].

The *HIPPO* signaling pathway is involved in cellular growth control, division, survival, as well as migratory and invasive behavior. Its uniqueness lies in the absence of an extracellular ligand or of a dedicated plasma membrane receptor, indicating that its activation is handled through crosstalk mechanism. In the context of iCCA, the HIPPO pathway dysfunction contributes to tumorigenesis and metastasis: its loss of function, which normally includes suppressing YAP/TAZ, can lead to their hyperactivation, which in turn promote cell proliferation and invasiveness. This mechanism has been extensively studied in various cancers, including BTC [160]. In addition, key HIPPO pathway proteins like Mst1 and Mst2 are required to maintain cellular homeostasis and prevent tumorigenesis, suggesting that dysfunction in these signaling molecules may promote this type of cancer. Inactivation of the HIPPO pathway can also affect the tumor microenvironment, favoring invasiveness and resistance to treatment through activation of YAP/TAZ [161]. Given this information, targeting the YAP/TAZ pathway could be a promising therapeutic strategy.

### 3.6. Sonic Hedgehog (SHH) Pathway

The Sonic Hedgehog (SHH) pathway is highly conserved in mammals throughout evolution, hinting at its importance critical cellular processes. It plays a recognized role in cellular metabolism, cell lineage differentiation, and the progressive regeneration of tissues and stem cells [162,163]. Thus, it is easy to understand how impairment of the SHH pathway can contribute to the development of diseases, and particularly to the onset of neoplasms [164].

Three different SHH ligands exist: Sonic Hedgehog (Shh), the predominant form in mammals, Indian Hedgehog (Ihh), present at a similar concentration to Shh, and Desert Hedgehog (Dhh), whose expression is mostly restricted to the gonads. These three proteins share the same morphogenic structure; however, they exhibit different tissue-specific distributions and dose-dependent efficacies [165].

Once secreted, SHH ligands interact with the transmembrane receptor PTCH1, which is rapidly internalized and degraded. This leads to the interruption of inhibition on another G-protein-coupled receptor, Smoothened (SMO), which culminates in the modulation of target gene transcription through the action of GLI transcription factors [165].

Each of these components can be deregulated, contributing to pathway dysfunction. Additionally, the complex bidirectional interactions between this pathway and others, such as RAS/RAF/MEK/ERK, PI3K/AKT, and TGF-β, can facilitate the bypassing of the SHH stepwise activation, leading to the direct regulation of downstream GLI and promoting cell proliferation and tumor transformation [166,167].

The SHH cascade has been shown to be essential for liver structural integrity, both during embryonic development and in tissue repair processes [168]. However, in adulthood, the SHH pathway remains largely quiescent due to the limited production of natural Hedgehog ligands by liver cells and the expression of inhibitory molecules, such as the Hedgehog-interacting protein (Hhip) produced by liver sinusoidal cells. This pathway can be reactivated in response to various stimuli, including chronic inflammation, which is a known risk factor for hepatic carcinogenesis [169].

In general, upregulation of the HH pathway is strongly correlated to oncogenic transformation and the development of iCCA, gallbladder carcinoma, hepatocellular carcinoma (HCC), and hepatoblastoma [170,171,172].

Focusing on iCCA, genetic alterations in key components of the SHH signaling pathway—such as GLI1, GLI2, GLI3, SMO, PTCH1/2, SUFU, and DHH—have been described. Notably, mutations in GLI3 and Desert Hedgehog are present in 4.6% and 5.6% of cases, respectively. An analysis of 50 BTC cases found overexpression of GLI1 and PTCH1 in 50% and 30% of cases, respectively, with SHH pathway overactivation detected in 50% of tumor cells [173].

In general, SHH hyperactivation correlates with a worse prognosis, as it is associated with lymph node involvement, high nuclear grade, intrahepatic metastasis, and shorter mPFS and mOS [174,175,176]. These findings have led to the hypothesis that SHH inhibitors could be incorporated into clinical practice to expand the pharmacological arsenal against CCA. Currently available SHH inhibitors can be categorized into two main groups: SMO inhibitors and GLI inhibitors.

The SMO inhibitor cyclopamine, either alone or in combination with other agents such as MAPK inhibitors, has demonstrated efficacy on inhibiting CCA growth and proliferation, preventing tumor shedding and invasion in xenograft models. However, its routine clinical application is severely limited due to poor oral bioavailability and severe adverse effects, which have precluded the development of clinical trials [171,177].

Vismodegib, another SMO inhibitor approved for the treatment of basal cell carcinoma, has also shown preclinical activity. However, the emergence of resistance mechanisms poses a challenge to long-term responses to this compound [178,179]. For this reason, new strategies and novel drugs are currently under investigation.

### 3.7. Wnt/B-Catenin Pathway

The Wnt/Beta-catenin pathway is a signalling pathway that is involved in several cellular processes, including regulation of cell growth, differentiation, and survival and in carcinogenesis of different types of cancers [180]. Activation of the Wnt pathway leads to the accumulation of β-catenin in the cytoplasm, which then translocates into the nucleus, where it interacts with transcription factors to promote the transcription of genes that favor cell proliferation [181]. The hyperactivation of the Wnt/β-catenin pathway is a key driver in the pathogenesis of iCCA, with increased β-catenin expression correlating with more aggressive tumor behavior [182].An increased mRNA expression of different Wnt ligands (Wnt3a, Wnt5a, Wnt7b and Wnt10a) has been demonstrated in CCA cells compared to normal tissue, possibly indicating their role as a marker of tumorigenesis. Moreover, a decrease in membranous expression of beta-catenin and conversely an increase in its nuclear localisation, were described as characteristics of iCCA tissues and were correlated to the presence of metastasis, vascular invasion and high grade of tumor [183]. The Wnt pathway is strongly interconnected with the Notch and Sonic Hedgehog pathways, which has implications for therapeutic interventions in cancers. There are significant challenges in targeting the Wnt pathway, including finding agents that are efficacious without damaging the system of normal somatic stem cell function in cellular repair and tissue homeostasis.

Similarly to HCC, the mutations of CTNNB1 and AXIN1 also promote CCA cell proliferation [184]. However, the frequency of mutations is different from HCC, i.e., AXIN1 (4%), and CTNBB1 (1.5%), respectively [180].

**Figure 2:**
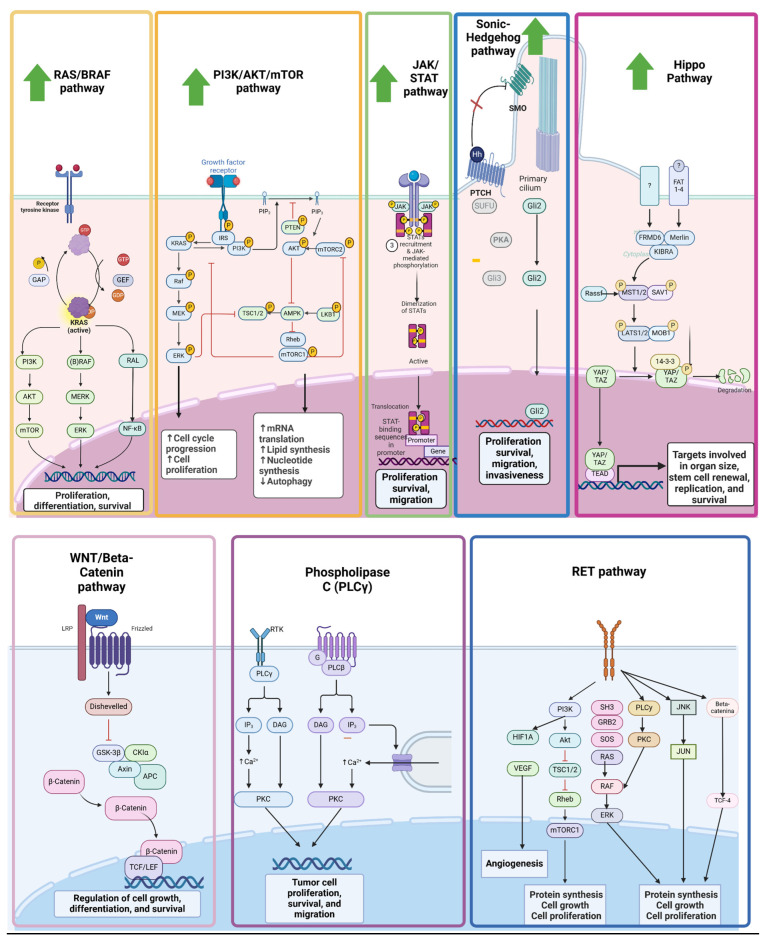
Pro-oncogenic pathway details. Upward arrows indicate upregulation of target protein.

We summarized pro-oncogenic pathway in Figure 2.

## 4. Tumor Suppressor Genes

### 4.1. TP53

Tumor Protein p53 (TP53) mutations are present in approximately 7% to 49% of iCCA cases, with prevalence varying significantly based on associated risk factors, such as liver fluke infection [185]. Notably, TP53 mutations are more frequently observed in patients with Opisthorchis viverrini infestation or those who are hepatitis B surface antigen (HBsAg) seropositive [78]. TP53 mutations often coexist with other genetic alterations, including KRAS mutations, and are associated with poor clinical outcomes [186].

In a Chinese study, a high prevalence of the R249S mutation in TP53 was noted. This is, a known aflatoxin-induced mutation., suggesting a possible environmental or dietary carcinogen exposure in Chinese iCCA patients [186].

**Figure 3:**
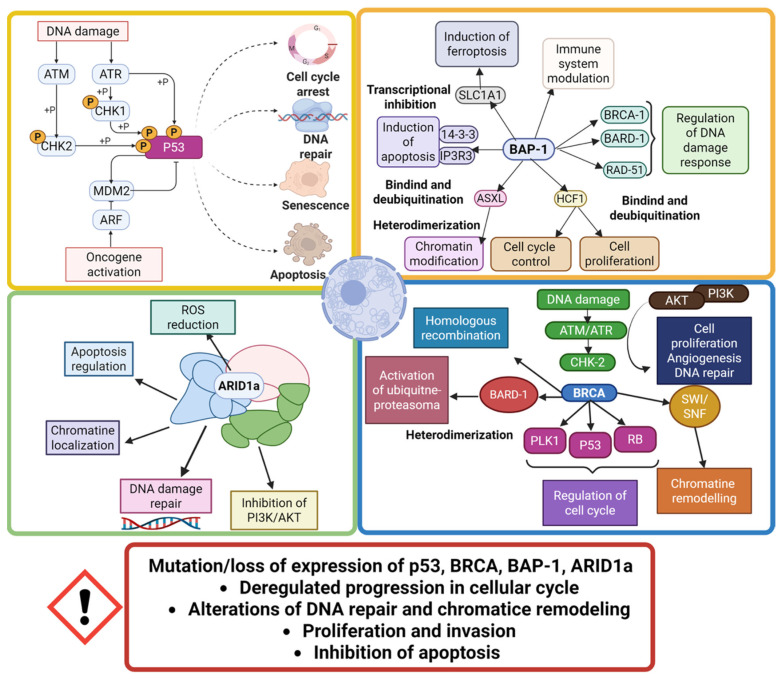
Tumor suppressor genes pathways details.

We summarized tumor suppressor genes pathways in Figure 3.

### 4.2. PTEN—CDKN2A

Recent studies have elucidated the involvement of tumor suppressor genes, such as PTEN (Phosphatase and Tensin Homolog) and CDKN2A (Cyclin-Dependent Kinase Inhibitor 2A), in the development and progression of different kinds of tumors. PTEN negatively regulates the PI3K/AKT pathway, which, as seen before, is essential for different cellular processes. Furthermore, PTEN loss was associated with increased tumorigenic potential, suggesting that this gene acts as a critical negative regulator of tumor growth in the bile duct epithelium, at least in murine models [187]. Still in mice, different studies have shown that liver-specific disruption of PTEN and activation of Kras caused rapid development of intrahepatic biliary epithelial proliferative lesions, which progress through dysplasia to invasive carcinoma. In contrast, Kras activation in combination with heterozygous PTEN deletion induced mixed carcinomas of liver (both iCCA and HCC), whereas Kras activation alone did not induce biliary tract neoplasm [188]. Thus, this data indicates the presence of a functional link between PTEN gene status, hepatobiliary cell differentiation, and ICCA, possibly indicating a new interesting target for drug development.

### 4.3. BRCA

BRCA1 (BReast CAncer gene 1) and BRCA2 (BReast CAncer gene 2) encode key proteins involved in homologous recombination (HR)-mediated DNA repair. Mutations in these genes result in impaired DNA damage repair, leading to genomic instability and an increased predisposition to malignancy. In iCCA, BRCA mutations are detected in approximately 4% of cases, highlighting a potential avenue for targeted therapeutic strategies [189].

Given their role in DNA repair deficiency, BRCA-mutated tumors exhibit increased sensitivity to DNA-damaging agents, including platinum-based chemotherapy and poly (ADP-ribose) polymerase (PARP) inhibitors. PARP inhibitors, such as olaparib, have shown efficacy in BRCA-mutated malignancies, and emerging evidence suggests a similar therapeutic potential in iCCA [190].

### 4.4. BAP-1

The ubiquitin–proteasome system is a protein degradation machinery essential for maintaining balanced protein turnover. The activity of this complex is counteracted by deubiquitinating enzymes (DUBs), which play a role in cell cycle progression, malignant transformation, and transcriptional regulation [191].

(BRCA1)-associated protein-1 (BAP1) is a tumor suppressor belonging to the DUB family [192]. Its main role involves chromatin remodeling, acting synergistically with other common enzymes such as ARID1A and PBRM1 [193]. Various BAP1 alterations, including germline or somatic mutations as well as epigenetic changes, have been implicated in the development of different cancer types [194,195].

In particular, germline mutations have been associated with a genetic cancer predisposition syndrome known as BAP1 tumor predisposition syndrome, which is primarily characterized by an increased lifetime incidence of basal cell carcinoma, renal cell carcinoma, uveal melanoma, mesothelioma, and iCCA [196]. This germline mutation follows an autosomal dominant inheritance pattern with high penetrance, as approximately 80% of carriers are expected to develop cancer during their lifetime [197]. Notably, in some cases, loss of the second allelic copy of the gene has been detected, suggesting a potential tumor suppressor role for BAP1 in accordance with the classic two-hit inactivation model [197].

Several studies have demonstrated that BAP1 downregulation is extremely common in iCCA and is associated with increased cellular proliferation, local tissue invasion, lymph node metastasis, advanced TNM stage, higher CA19-9 levels, and mPFS and mOS in preclinical studies. These effects are likely mediated through the modulation of other intracellular pathways, such as extracellular signal-regulated kinase 1/2 (ERK1/2) and c-Jun N-terminal kinase (JNK)/c-Jun signaling [198].

It is estimated that BAP1 mutations occur in 15–35% of BTC, with the highest frequency observed in iCCA. While BAP1 has been suggested as a negative prognostic factor in CCA, the available literature remains inconclusive, with conflicting studies reporting no clear impact of BAP1 mutations on OS and PFS [199,200]. Nonetheless, BAP1 remains one of the most promising drug targets to be further explored.

### 4.5. SWI/SNF Pathway

SWI/SNF is a chromatin remodeling complex that acts as a tumor suppressor, regulating genic expression through the modulation of chromatin envelopment and accessibility to transcriptional machinery [201]. Globally, epigenetic alteration of the entire complex can be found in 20% of cancers [202]. One of the most important and clinically relevant complex subunits is AT-rich interactive domain-containing protein 1A (ARID1A), that, together with PBRM-1, is mutated in 17–19% of iCCA. The prognostic impact of these alterations has not yet been fully elucidated, however some papers report a possible negative impact on survival [203,204]. Focusing on ARID1a, it has gained interest in the scientific community since its alterations have been linked to regulation of the hepatic microenvironment and metastasisation processes [205].

ARID1a-deficient carcinomas can represent a potential target for drugs such as PARP inhibitors, which have demonstrated to be effective in the presence of these mutations [206].

Finally, several co-mutated genes have been observed in ARID1a altered cancers. For example, some authors have proved the existence of a cooperative role of inactivating mutation of ARID1a and activating mutations of KRAS through the attenuation of TGF-β-SMAD pathway [207]. In addition, the presence of SWI/SNF complex impairment is often accompanied by hyperactivation of the PI3K pathway, thus PI3K/mTOR/AKT inhibitors could also be potentially used in this setting [208]. Lastly, an increased expression in PD-1/PD-L1 has been identified in ARID1a-mutant tumors, possibly indicating an interesting line of research regarding the presence of these mutations and the efficacy of immunotherapies in this subset of patients [209].

## 5. Metabolic and Other Pathways

### 5.1. S1P

The S1P (sphingosine-1-phosphate) pathway regulates numerous biological processes, including cell proliferation, survival and migration [210,211]. A central element of this pathway is the enzyme sphingosine kinase 1 (SPHK1), which catalyzes the production of S1P. The overexpression of SPHK1 has been observed in many types of cancer cells. The S1P pathway has been demonstrated to be involved in CCA progression, as reflected by increased tumor growth and associated malignant biliary obstruction in relation to a progressive increase in SPHK1 expression in an orthotopic rat CCA model [212]. Several studies have also demonstrated that S1PR2 is highly expressed in rat and human CCA cells, as well as in human CCA tissues. Taurocholate (TCA)-mediated CCA cell proliferation, migration, and invasion were significantly inhibited by JTE-013, a chemical antagonist of S1PR2, or by lentiviral short hairpin RNA silencing of S1PR2 [145].

### 5.2. FOXO1

Forkhead box transcription factors (FOXO) family proteins are growth factors and stress-regulated transcription factors that normally reside in the nucleus of quiescent or growth factor deprived cells. In the presence of cell growth factors, FOXO proteins are relocated to the cytosol and eventually subjected to degradation via the ubiquitin–proteasome pathway. In the absence of the cellular survival drive of growth factors, FOXO proteins translocate to the nucleus and upregulate a series of target genes, thereby promoting cell cycle arrest, stress resistance, and apoptosis [213]. FOXO transcription factors have been reported to play pivotal roles in tumorigenesis and drug resistance. Importantly, FoxO3 deficiency strongly potentiated tumor formation in nude mice and it rendered CCA xenografts resistant to cisplatin-induced cell death by activating Nrf2. Additionally, clinical CCA samples displayed FoxO3-Keap1 down-regulation and Nrf2 hyperactivation, hinting at their important roles in CCA development [214]. In addition, it was reported that FOXO1 regulates the basal and serum starvation (SS)-induced autophagy, thus it representing a potential therapeutic target in iCCA via regulation of autophagy, oxidative stress and mitochondrial dysfunction [215].

**Figure 4:**
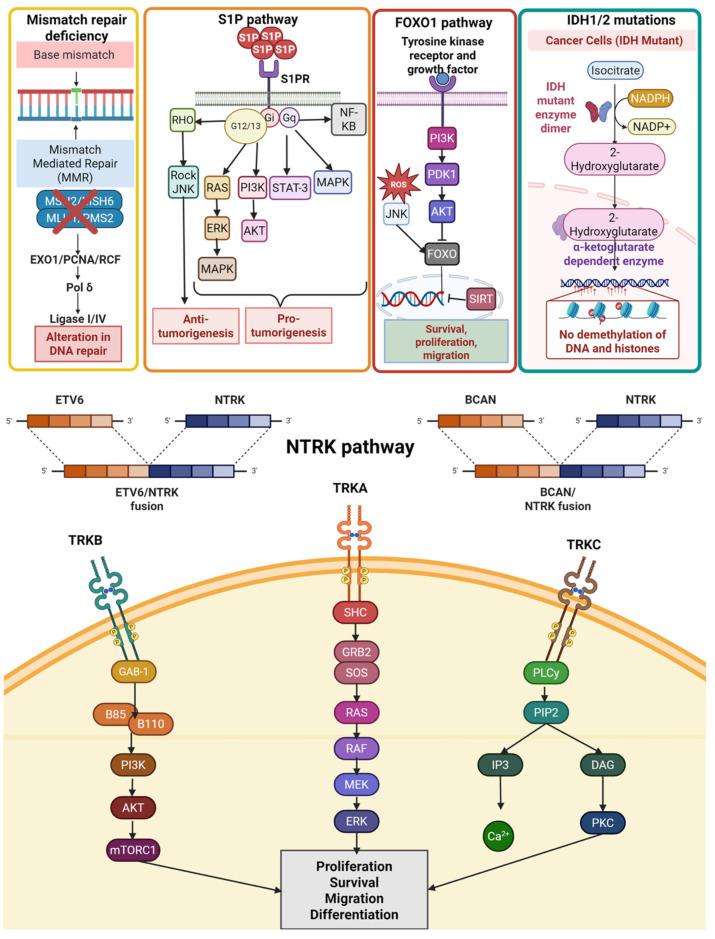
Metabolic and other pathway details. Red “X” in the figure indicates alterations in DNA mismatch repair genes.

We summarized metabolic and other pathways in Figure 4.

### 5.3. IDH1/IDH2 Mutations

Isocitrate dehydrogenase (IDH) represents a crucial metabolic enzyme in cellular respiration within the tricarboxylic acid cycle. There are three main IDH subtypes, with IDH1 and IDH2 being particularly relevant for catalyzing the NADP^+^-dependent oxidative decarboxylation of isocitrate to α-ketoglutarate (α-KG) and CO_2_ [216]. Recurrent somatic mutation gain of function mutations typically occur at a single amino acid residue in both IDH1 (arginine 132) and IDH2 (arginine 172 or arginine 140) and disrupt the normal catalytic activity of IDH1/2. As a result, α-KG is increasingly converted into D-2 hydroxyglutarate (D2HG), an oncometabolite that promotes tumor growth and metastasis through various mechanisms, including DNA methylation and VEGFR activation [217]. IDH mutations are frequently observed in several rare malignancies, including iCCA where the frequency is particularly high compared to other BTC. In a small cohort of iCCA patients, the percentage of IDH mutations has been reported to reach up to 25%, although other papers report a lower incidence of 15–20% [218,219]. As mentioned, these mutations lead to elevated levels of the oncometabolite D2HG, which can serve as a surrogate biomarker for IDH-mutant iCCA when detected in tissue or blood: increased D2HG levels are associated with higher DNA CpG methylation and altered histone methylation, resulting in epigenetic modifications that impair the differentiation of iCCA cells. Furthermore, IDH mutations influence hypoxia signaling, collagen processing, and epithelial-to-mesenchymal transition (EMT) by upregulating ZEB1 and downregulating miR-200. Additionally, IDH1/2 mutations frequently interact with tyrosine kinase and MAPK-dependent signaling pathways. IDH1 and IDH2 mutations are mutually exclusive with NRAS/KRAS and FGFR mutations but may coexist with BAP1 mutations [220]. Several selective inhibitors targeting IDH have been developed: among them, ivosidenib represents the gold standard second-line treatment for patients harboring IDH1 mutations thanks to the phase III ClarIDHy trial [221]. Currently, multiple phase I and II clinical trials are evaluating IDH1/2 inhibitors for CCA, as summarized in the Supplementary Table S1.

### 5.4. Mismatch Repair Deficiency Pathway

The mismatch repair (MMR) pathway plays a crucial role in maintaining genomic stability by correcting errors during DNA replication: its dysfunction leads to microsatellite instability (MSI), which is associated with the accumulation of mutations and oncogenesis. In CCA, MSI or MMR deficiency (MMR-d) can result from mutations in genes such as MLH1, MSH2, MSH6, and PMS2. The MMR proteins can be inactivated through germline (Lynch syndrome) or sporadic mutations, or they can be silenced through promoter hypermethylation, typically of the MLH1 gene 1 and 2 [222]. These changes lead to hypermutation during DNA replication, leading to the development of malignancies, most notably colorectal and uterine carcinomas. These changes also increase the neoantigen load of the tumor, allowing for more susceptibility to immunotherapies because of the increased inflammation surrounding these tumors [223]. Approximately 6% of BTC, seems to have MSI based on MMR IHC loss, with 10% being iCCA and 5% eCCA. [224]. Studies have shown that in CCA cases that are associated with chronic liver diseases or parasitic infections by Opisthorchis Viverrini, MMR deficiency is observed and that the presence of MSI correlates with better survival outcomes [225].

Only a limited number of studies have been performed to assess the efficacy of immune-checkpoint inhibitors in BTC, with available clinical data being mainly limited to sub-analyses of basket trials and small single-arm trials. In cases of MMR-d BTC, treatment with ICIs has demonstrated clinical benefit. In the prospective, non-randomised, phase II KEYNOTE-158 trial, 22 patients with CCA and MSI-H/MMR-d were treated with pembrolizumab: an ORR of 40.9% was achieved with a mPFS of 4.2 months and mOS of 24.3 months, supporting the use of pembrolizumab in patients who lack other therapeutic options [226]. Beyond immune checkpoint blockade, other therapeutic strategies are being explored to target MMR-deficient CCA. For example, combining immune checkpoint inhibitors with conventional chemotherapy might improve outcomes, as chemotherapy can increase the mutational load, potentially enhancing the effectiveness of immunotherapy in MMR-deficient tumors [227]. Additionally, targeting other DNA repair pathways, such as with PARP inhibitors, could be beneficial in combination with immunotherapy in MMR-deficient CCA [228].

## 6. Conclusions

Intrahepatic cholangiocarcinoma (iCCA) is a biologically and genomically heterogeneous malignancy in which multiple oncogenic pathways converge to drive tumor initiation, progression, and therapeutic resistance. Over the past decade, comprehensive molecular profiling has improved our understanding of iCCA pathogenesis, allowing the identification of biologically distinct subgroups and accelerating the integration of precision oncology into clinical practice. Among the currently actionable alterations, FGFR2 fusions and IDH1 mutations represent the most robustly validated molecular targets, and their respective inhibitors have already entered the therapeutic algorithm significantly leading to better outcomes. Likewise, the rapid evolution of HER2-directed therapies—including bispecific antibodies and antibodies–drug conjugates—and the tumor-agnostic approval of NTRK and RET inhibitors, mark an additional step toward a genomically driven therapeutic paradigm. Despite these advances, the majority of patients with iCCA remain without effective targeted options. The RAS/RAF/MEK/ERK and PI3K/AKT/mTOR cascades represent key signaling routes whose dysregulation contributes to tumor growth, survival, and therapeutic resistance in tumors harboring alterations such as KRAS, BRAF, PIK3CA, or PTEN. Moreover, the Notch, TGF-β, Hippo/YAP, and Hedgehog pathways, which orchestrate stemness, epithelial–mesenchymal transition, and microenvironmental remodeling, are emerging as critical regulators of aggressiveness and therapeutic escape, with several agents investigated through early-phase clinical trial. Additionally, alterations in chromatin-remodeling genes—such as ARID1A, BAP1, and other SWI/SNF complex components—suggest opportunities for exploiting synthetic lethality approaches, including PARP inhibition, PI3K-pathway blockade, and immunotherapy-based combinations. Integration of these molecular insights into clinical practice will require broad and systematic adoption of next-generation sequencing, coupled with the design of biomarker-driven trials capable of capturing the full complexity of iCCA biology. Ultimately, the convergence of genomics, tumor microenvironment characterization, and pathway-directed therapeutics has the potential to transform iCCA from a uniformly lethal disease into a condition increasingly managed through individualized, mechanism-based strategies.

## Data Availability

No new data were created or analyzed in this study. Data sharing is not applicable to this article.

## Supplementary Materials

The following supporting information can be downloaded at: https://www.mdpi.com/article/10.3390/ijms262411961/s1. References [229,230,231,232,233,234,235,236,237,238,239,240,241,242,243,244,245,246,247,248,249,250,251,252,253,254,255,256,257,258,259,260,261,262,263,264,265,266,267,268,269] are cited in the Supplementary Materials.

## Abbreviations

The following abbreviations are used in this manuscript:

ADAM	A disintegrin and metalloproteinase
ADP	Adenosine diphosphate
AKT	Protein kinase B
ALDH1A3	Aldehyde Dehydrogenase 1 Family Member A3
ALK	Anaplastic lymphoma kinase
AP-1	Activator protein 1
ARID1A	AT-Rich Interaction Domain 1A
AT	Ataxia Telangiectasia
ATR	Ataxia Telangiectasia Rad3-related protein
AXIN1	Axis inhibition protein 1
BAF	BRG1/BRM associated factor
BAFF	B cell activating factor
BAP1	BRCA1 associated protein-1
BP1	Homebox protein 1
BRCA	Breast cancer gene
BRG1	Brahma-related gene 1
BTC	Biliary tract cancer
C-MYC	Cellular MYC
CA19-9	Carbohydrate antigen 19-9
CCA	Cholangiocarcinoma
CCL5	Chemokine (C-C motif) ligand 5
CD24	Cluster of differentiation 24
CDKN2A	Cyclin-dependent kinase inhibitor 2A
CNS	Central nervous system
COX2	Cyclooxygenase-2
CTNBB1	Catenin beta-1
CXCL12	Chemokine (C-X-C motif) ligand 12
CXCR4	C-X-C chemokine receptor type 4
D2HG	D-2 hydroxyglutarate
DHH	Desert Hedgehog
DNA	Deoxyribonucleic acid
DUB	Deubiquitinating enzyme
eCCA	Extrahepatic cholangiocarcinoma
EGF	Epidermal growth factor
EGFR	Epidermal growth factor receptor
EMT	Epithelial-to-mesenchymal transition
ERBB2	Human Epidermal growth factor 2
ERK	Extracellular signal-regulated kinase
ESMO	European Society for Medical Oncology
FGF	Fibroblast Growth Factor
FGFR	Fibroblast Growth Factor Receptor
FOXO	Forkhead box transcription factors
GBC	Gallbladder cancer
GH	Growth hormone
GLI	GLI family zinc finger transcription factors
GOF	Gain-of-function
GTP	Guanosine triphosphate
HCC	Hepatocellular carcinoma
HES	Enhancer of split
HGF	Hepatocyte growth factor
HH	Hedgehog
HIF	Hypoxia-inducible factor
HR	Homologous recombination
iCCA	intrahepatic cholangiocarcinoma
IDH	Isocitrate Dehydrogenase
IGF	Insulin-like growth factor
IGFR	Insulin-like growth factor receptor
IHC	Immunohistochemistry
IL	Interleukin
IMR-1	Inhibitor of Mastermind Recruitment-1
JAG	Jagged
JAK	Janus Kinase
JNK	c-Jun N-terminal kinase
KCSG	Korean Cancer Study Group
KRAS	Kirsten Rat Sarcoma viral oncogene homolog
LATS	Large Tumor Suppressor
LIF	Leukemia inhibitory factor
LOF	Loss-of-function
MAML-1	Mastermind-like proteins
MAP	Mitogen-activated protein
MAPK	Mitogen-activated protein kinase
MMR	Mismatch repair
MSI	Microsatellite instability
MTOR	Mammalian target of rapamycin
MUC4	Mucin 4
NRAS	Neuroblastoma RAS viral oncogene homolog
NADP	Nicotinamide adenine dinucleotide phosphate
NCCN	National Comprehensive Cancer Network
NEJM	New England Journal of Medicine
NF-kB	Nuclear factor kappa B
NGS	Next Generation Sequencing
NICD	Notch intracellular domain
NOTCH	Notch receptor
NTRK	Neurotrophic Tyrosine Receptor Kinase
ORR	Objective response rate
OS	Overall survival
PARP	Poly(ADP-ribose) polymerase
PBRM-1	Polybromo 1
PD-1	Programmed cell death protein 1
PD-L1	Programmed cell death protein ligand 1
PDGF	Platelet-derived growth factor
PDGFR	Platelet-derived growth factor receptor
PDK1	Phosphoinositide-dependent kinases
PFS	Progression Free Survival
PGF	Placental growth factor
PI3K	Phosphatidylinositol 3-kinase
PIAS	Protein inhibitors of activated STAT proteins
PIP2	Phosphatidylinositol-4,5-bisphosphate
PIP3	Phosphatidylinositol-3,4,5-bisphosphate
PTCH1	Protein patched homolog 1
PTEN	Phosphatase and tensin homolog
RBP-J	Recombination Signal Binding Protein for Immunoglobulin Kappa J
RET	REarranged during Transfection
RNA	Ribonucleic acid
ROS1	c-ros oncogene 1
RTK	Receptor tyrosine kinase
S1P	Sphingosine-1-phosphate
SDF-1	Stromal-Derived Factor-1
SHH	Sonic Hedgehog
SOCS	Suppressors of cytokine signaling
SPHK1	Sphingosine kinase 1
SREBP1	Sterol regulatory element-binding proteins
STAT	Signal Transducer and Activator of Transcription
SUFU	Suppressor of fused homolog
SWI/SNF	Switch/Sucrose non fermentable
TAZ	Transcriptional co-activator with PDZ-binding motif
TCA	Taurocholate
TGF	Transforming growth factor beta
TKI	Tyrosine kinase inhibitor
TNF	Tumor necrosis factor
TP53	Tumor Protein p53
TRAF6	TNF receptor-associated factor 6
TRAIL	Tumor necrosis factor-related apoptosis inducing ligand
TSC1	Tuberous sclerosis complex
VEGF	Vascular endothelial-derived growth factor
VEGFR	Vascular endothelial growth factor receptor
YAP	Yes-associated protein
ZEB1	Zinc finger E-box-binding homeobox 1
