# Supplementary material for: Molecular Alterations and Pathways in Intrahepatic Cholangiocarcinoma: Available Evidence and New Perspectives
Source: Int J Mol Sci. 2025 Dec 11;26(24):11961. doi: 10.3390/ijms262411961 (PMC12732427; doi:10.3390/ijms262411961)
Supplement: Supplementary file 1 [file ijms-26-11961-s001.zip › ijms-3988494-supplementary.pdf]

Table S1. Summary of targeted therapy clinical trials in patients with intrahepatic cholangiocarcinoma.

	Target	Drug Name	Study Name/PMID/NCT	Phase	Year	Author	Therapy Line	Approval
1	ALK	Entrectinib	STARTRK-2 NCT02568267	II	ONGOING	/	II and beyond	NO
		Crizotinib	AcSè trial NCT02034981	II	2023	/	II and beyond	NO
2	BAP-1/ ARID1a	Niraparib	UF-STO-ETI-001 trial NCT03207347	II	2023	/	II and beyond	NO
3	BRAF	Vemurafenib	26287849	II	2015	Hyman DM et al. [1]	II and beyond	NO
		Dabrafenib + Trametinib	ROAR trial 37059834	II	2023	Subbiah V et al. [2]	II and beyond	YES (FDA)
		Dabrafenib + Pazopanib	NCT01713972	I	2016	Shah M et al. [3]	II and beyond	NO
4	BRCA	Veliparib (ABT-888)+ Cisplatin + Gemcitabine	NCT01282333	I	2013	/	II and beyond	NO
5	EGFR	Cetuximab + Gemcitabine	23975665	II	2013	Borbath I. et al. [4]	I	NO
		Cetuximab + Gemcitabine + Oxaliplatin	BINGO trial	II	2014	Malka D. et al. [5]	I	NO
		Panitumumab + Cisplatin + Gemcitabine	PICCA trial NCT01320254	II	2917	/	I	NO
		Varlitinib ASLAN001	NCT02609958	II	2018	/	II and beyond	NO
6	FGFR	ARQ087 (derazantinib)	30420614	I/II	2019	Mazzaferro V et al. [6]	II and beyond	NO
			FIDES-01 NCT03230318	II	2022	Milind M et al. [7]	II and beyond	NO
		JNJ-42756493 (erdafitinib)	NCT02699606 39138436	IIa	2024	Park JO et al. [8]	II and beyond	NO
		TAS-120	FOENIX-101	II	2019	Goyal L. et al. [9]	II and beyond	NO
		BGJ398 (infigratinib)	29182496	II	2018	Javle M. et al. [10]	II	NO
			PROOF-301 trial 32580579	III	2020	Makawita S et al [11]	I	NO

		Pemigatinib (INCB054828)	FIGHT-202 32203698	II	2020	Abou-Alfa GK et al [12]	II and beyond	YES (EMA, FDA, AIFA)
			FIGHT-302	III	2020	Bekaii-Saab TS et al. [13]	I	NO
		Pemigatinib + Afatinib	NCT06302621	I	ONGOING	/	II and beyond	NO
		BAY1163877 (Rogaratinib)	31405822	I	2019	Schuler M et al. [14]	II and beyond	NO
		Debio 1347-101	NCT01948297	I	2020	Cleary JM et al. [15]	II and beyond	NO
		Ponatinib	NCT02265341	II	2018	De Leon T et al. [16]	II and beyond	NO
			NCT02272998	II	2022	Roychowdhury S et al. [17]	II and beyond	NO
7	HER2	Trastuzumab + Pertuzumab	My Pathway 34339623	IIa basket trial	2021	Javle et al. [18]	II and beyond	NO
		Zanidatamab	NCT02892123	I	2022	Meric Bernstam et al. [19]	II and beyond	NO
			HERIZON-BTC-01 37276871	IIb	2023	Harding JJ et al. [20]	II and beyond	NO
		Zanidatamab + cisplatin and gemcitabine ± durvalumab or pembrolizumab vs SoC	HERIZON-BTC-302 NCT06282575	III	ONGOING	/	I	NO
		Trastuzumab + tucatinib	SGNTUC-019 trial 37751561	II basket study	2023	Nakamura Y et al. [21]	II and beyond	NO
		Trastuzumab + FOLFOX	KCSG-HB19-14 trial 36328033	II	2022	Lee et al. [22]	II-III	NO
		Trastuzumab + cisplatin + gem-citabine	TAB trial 37944079	II	2024	Ostwal V et al. [23]	I	NO
		Tucatinib + trastuzumab + FOL-FOX/CAPOX ± pembrolizumab	NCT04430738	Ib/II	ONGOING	/	I	NO
		Trastuzumab Deruxtecan	HERB trial 35510484	II	2022	Ohba A et al [24]	II and beyond	YES (AIFA)
			DESTINY-PanTumor02 trial	II	2024	Meric-Bernstam F et al. [25]	II and beyond	

		Neratinib	SUMMIT trial 36746967	II basket trial	2023	Harding JJ et al [26]	II and beyond	NO
		GQ1001	NCT04450732	I	ONGOING	/	II and beyond	NO
		DB-1303/BNT323	NCT05150691	I/IIa	ONGOING	/	II and beyond	NO
		Pertuzumab zuvotolimod (SBT6050) */- Pembrolizumab / Cemiplimab	NCT04460456	I	ONGOING	/	II and beyond	NO
		ISAC BDC-1001 + /- Nivolumab	NCT04278144	I	ONGOING	/	II and beyond	NO
		CR-0508 +/- Pembrolizumab	NCT04660929	I	ONGOING	/	II and beyond	NO
		IAH0968 + gemcitabine + cis-platin	NCT05991518	I/II	ONGOING	/	II and beyond	NO
		Disitamab vedotin (RC48-ADC)	NCT04329429	II	ONGOING	/	II and beyond	NO
		Disitamab vedotin (RC48-ADC) + Envolizumab	NCT05417230	II	ONGOING	/	I	NO
		Disitamab vedotin (RC48-ADC) + zimberelimab	RIGHT trial NCT05540483	II	ONGOING	/	II and beyond	NO
		Trastuzumab + Pembrolizumab + Cisplatin + Gemcitabine	TRAP-BTC trial NCT06178445	II	ONGOING	/	I	NO
		Trastuzumab + Nivolumab + Cisplatin + Gemcitabine	HERBOT trial NCT05749900	Ib/II	ONGOING	/	I	NO
		KN026 + Capecitabine + Oxali-platin ± KN-046	NCT05985707	II	ONGOING	/	I	NO
8	HGF/ c MET	Merestinib + Cisplatin * Gem-citabine	NCT02711553	II	ONGOING	/	I	NO
		Cabozantinib	28192597	II	2017	Goyal L. et al. [27]	II and beyond	NO
9	IDH	Ivosidenib	ClarIDHy 32416072	III	2020	Abou-alfa GK. et al. [28]	II and beyond	YES (EMA, FDA, AIFA)
		Dasatinib	NCT02428855	II	2020	/	II and beyond	NO
		Olaparib + Durvalumab	NCT03991832	II	ONGOING	/	II and beyond	NO
		HMPL-306	NCT04762602	I	ONGOING	/	II and beyond	NO
		Olaparib + AZD6738	NCT03878095	II	ONGOING	/	II and beyond	NO
		Enasidenib (AG-221)	NCT02273739	II	2021	/	II and beyond	NO

		IDH305	NCT02381886	I	ONGOING	/	III	NO
10	JAK/STAT	Tofacitinib + LMB-100	NCT04034238	I	ONGOING	/	II and beyond	NO
11	K-RAS	Sotorasib	NCT03600883 (CodeBreak-100)	I/II	ONGOING	/	II and beyond	NO
		DCC-3116	NCT04892017	I/II	ONGOING	/	II and beyond	NO
12	MEK	Selumetinib (+ Gemcitabine / Cisplatin)	ABC-04 study	Ib	2016	Bridgewater J et al. [29]	I	NO
		MEK162 (+ Capecitabine)	31312030	Ib	2019	Kim JW et al. [30]	II and beyond	NO
		Trametinib	SWOG S1310 32234665	II	2020	Kim RD et al. [31]	II	NO
			29121415	IIa	2018	Ikeda M et al. [32]	II	NO
		Refametinib	NCT02346032	II	2017	/	II	NO
		Binimetinib + gemcitabine	30563938	I/II	2019	Lowery MA et al. [33]	I	NO
13	NOTCH	LY3039478 + other drugs	NCT02784795	I	2020	/	II and beyond	NO
14	NTRK	Entrectinib	NCT02568267	II	ONGOING	/	II and beyond	YES (EMA, FDA, AIFA)
			ALKA-372-001, STARTRK-1, and STARTRK-2 31838007	I/II	2020	Doebele RC. et al. [34]	II and beyond	
		Larotrectinib	NAVIGATE NCT02576431	II	ONGOING	/	II and beyond	YES (EMA, FDA, AIFA)
15	PDGFR	Pazopanib + GSK1120212	NCT01438554	I	2018	/	I	NO
		Regorafenib	NCT02053376	II	2019	Sun W. et al. [35]	II	NO
16	PI3K/ mTOR/ AKT	Sirolimus	18307022	II	2007	Rizell et al. [36]	II and beyond	NO
			28685070	II	2017	Jung KS et al. [37]	II and beyond	NO
		Sirolimus + Cisplatin + Gem-citabine	NCT01888302	I	2016	/	Adiuvante	NO
		Everolimus	25145431	I	2014	Verzoni E. et al. [38]	II and beyond	NO
			ITMO study 24827133	II	2014	Buzzoni R. et al. [39]	II and beyond	NO
			RADiChol Study 29527009	II	2018	Lau DK. er al. [40]	I	NO
			28330462	II	2017	Kim ST et al. [41]	II and beyond	NO

			CRAD001T NCT01525719	II	2012	/	I	NO
		Everolimus + Gemcitabine + Cisplatin	24740268	I	2014	Costello BA. et al. [42]	I and beyond	NO
		Everolimus +Gem (cohort I) or CisGem (cohort II/III)	NCT00949949	I	2017	/	II and beyond	NO
		Everolimus + Gemcitabine + Oxaliplatin	NCT02836847	II	2018	/	I	NO
		Buparlisib	22162589	I	2012	Bendell JC. et al. [43]	II and beyond	NO
		Buparlisib + mFOLFOX6	26490655	I	2015	McRee AJ. et al. [44]	II and beyond	NO
		BAY80-6946 (Copanlisib) ±Gem or CisGem	29348486	I	2018	Kim RD. et al. [45]	II and beyond	NO
		MK2206	26161813	II	2015	Ahn DH. et al. [46]	II and beyond	NO
		LY2584702 + Erlotinib vs Everolimus	24456794	Ib	2014	Hollebecque A. et al. [47]	II and beyond	NO
		LY2780301	25902900	I	2015	Azaro A. et al. [48]	II and beyond	NO
		LY2780301 + Gemcitabine	28750271	Ib	2017	Angevin E. et al. [49]	II and beyond	NO
		Copanlisib + Cisplatin + Gemcitabine	33289918	II	2021	Tan ES. et al. [50]	I	NO
		Target agents according to tumor profile, among which<br>I: PIK3CA mut, no RAS mut or PTEN loss: taselisib<br>L: mTOR mut: sapanisertib<br>N: PTEN mut or deleted or expressed: GSK2636771<br>P: PTEN loss GSK2636771<br>Y: AKT mut: AZD5363	MATCH Screening Trial NCT02465060	II	ONGOING	/	II and beyond	NO
		Capecitabine + varlitinib/placebo	TreeTopp Trial 34922298	II	2022	Javle MM. et al. [51]	II	NO
17	PTEN	Bortezomib	NCT03345303	III	2017	/	III	NO
18	RET	Selpercatinib	Libretto-001 NCT03157128	I/II	ONGOING	/	II and beyond	NO

References

1. Hyman, D.M.; Puzanov, I.; Subbiah, V.; Faris, J.E.; Chau, I.; Blay, J.-Y.; Wolf, J.; Raje, N.S.; Diamond, E.L.; Hollebecque, A.; et al. Vemurafenib in Multiple Nonmelanoma Cancers with BRAF V600 Mutations. *N. Engl. J. Med.* **2015**, *373*, 726–736. https://doi.org/10.1056/NEJMoa1502309. Erratum in *N. Engl. J. Med.* **2018**, *379*, 1585.
2. Subbiah, V.; Lassen, U.; Élez, E.; Italiano, A.; Curigliano, G.; Javle, M.; de Braud, F.; Prager, G.W.; Greil, R.; Stein, A.; et al. Dabrafenib plus Trametinib in Patients with BRAFV600E-Mutated Biliary Tract Cancer (ROAR): A Phase 2, Open-Label, Single-Arm, Multicentre Basket Trial. *Lancet Oncol.* **2020**, *21*, 1234–1243. https://doi.org/10.1016/S1470-2045(20)30321-1.
3. Shah, M. NCT01713972—Dabrafenib and Pazopanib Hydrochloride in Treating Patients With Advanced Malignant Tumors. Available online: https://clin.larvol.com/trial-detail/NCT01713972 (accessed on 29 October 2025).
4. Borbath, I.; Ceratti, A.; Verslype, C.; Demols, A.; Delaunoit, T.; Laurent, S.; Deleporte, A.; Vergauwe, P.; Van Maanen, A.; Sempoux, C.; et al. Combination of Gemcitabine and Cetuximab in Patients with Advanced Cholangiocarcinoma: A Phase II Study of the Belgian Group of Digestive Oncology. *Ann. Oncol.* **2013**, *24*, 2824–2829. https://doi.org/10.1093/annonc/mdt337.
5. Malka, D.; Cervera, P.; Foulon, S. Gemcitabine and Oxaliplatin with or without Cetuximab in Advanced Biliary-Tract Cancer (BINGO): A Randomised, Open-Label, Non-Comparative Phase 2 Trial. *Lancet Oncol.* **2014**, *15*, 819–828. https://doi.org/10.1016/S1470-2045(14)70212-8.
6. Mazzaferro, V.; El-Rayes, B.F.; Droz Dit Busset, M. Derazantinib (ARQ 087) in Advanced or Inoperable FGFR2 Gene Fusion-Positive Intrahepatic Cholangiocarcinoma. *Br. J. Cancer* **2019**, *120*, 165–171. https://doi.org/10.1038/s41416-018-0334-0.
7. Basilea Pharmaceutica. NCT03230318—Derazantinib in Subjects With FGFR2 Gene Fusion-, Mutation- or Amplification- Positive Inoperable or Advanced Intrahepatic Cholangiocarcinoma (FIDES-01). Available online: https://clinicaltrials.gov/study/NCT03230318 (accessed on 29 October 2025).
8. Park, J.O.; Feng, Y.-H.; Su, W.-C. Erdafitinib in Asian Patients with Advanced Solid Tumors: An Open-Label, Single-Arm, Phase IIa Trial. *BMC Cancer* **2024**, *24*, 1006. https://doi.org/10.1186/s12885-024-12584-0.
9. Bahleda, R.; Meric-Bernstam, F.; Goyal, L. Phase I, First-in-Human Study of Futibatinib, a Highly Selective, Irreversible FGFR1–4 Inhibitor in Patients with Advanced Solid Tumors. *Ann. Oncol.* **2020**, *31*, 1405–1412. https://doi.org/10.1016/j.annonc.2020.06.018.
10. Javle, M.; Lowery, M.; Shroff, R.T. Phase II Study of BGJ398 in Patients with FGFR-Altered Advanced Cholangiocarcinoma. *J. Clin. Oncol.* **2018**, *36*, 276–282. https://doi.org/10.1200/JCO.2017.75.5009.
11. Makawita, S.; Abou-Alfa, G.K.; Roychowdhury, S. Infigratinib in Patients with Advanced Cholangiocarcinoma with FGFR2 Gene Fusions/Translocations: The PROOF 301 Trial. *Future Oncol.* **2020**, *16*, 2375–2384. https://doi.org/10.2217/fon-2020-0299.
12. Vogel, A.; Sahai, V.; Hollebecque, A.; Vaccaro, G.; Melisi, D.; Al-Rajabi, R.; Paulson, S.; Borad, M.; Gallinson, D.; Murphy, A.; et al. O-2 Pemigatinib for Previously Treated Locally Advanced or Metastatic Cholangiocarcinoma: Final Results from FIGHT-202. *Ann. Oncol.* **2022**, *33*, S379. https://doi.org/10.1016/j.annonc.2022.04.443.
13. Bekaii-Saab, T.S.; Valle, J.W.; Van Cutsem, E. FIGHT-302: First-Line Pemigatinib vs Gemcitabine Plus Cisplatin for Advanced Cholangiocarcinoma with FGFR2 Rearrangements. *Future Oncol.* **2020**, *16*, 2385–2399. https://doi.org/10.2217/fon-2020-0429.
14. Schuler, M.; Cho, B.C.; Sayehli, C.M. Rogaratinib in Patients with Advanced Cancers Selected by FGFR mRNA Expression: A Phase 1 Dose-Escalation and Dose-Expansion Study. *Lancet Oncol.* **2019**, *20*, 1454–1466. https://doi.org/10.1016/S1470-2045(19)30412-7.
15. Debiopharm International. NCT01948297—Debio 1347-101 Phase I Trial in Advanced Solid Tumours With Fibroblast Growth Factor Receptor (FGFR) Alterations. Available online: https://clinicaltrials.gov/study/NCT01948297 (accessed on 29 October 2025).
16. Mayo Clinic. NCT02265341—Ponatinib Hydrochloride in Treating Patients With Advanced Biliary Cancer With FGFR2 Fusions. Available online: https://clinicaltrials.gov/study/NCT02265341 (accessed on 29 October 2025).

17. Roychowdhury, S. NCT02272998—Ponatinib for Patients Whose Advanced Solid Tumor Cancer Has Activating Mutations Involving the Following Genes: FGFR1, FGFR2, FGFR3, FGFR4, RET, KIT. Available online: https://clinicaltrials.gov/study/NCT02272998 (accessed on 29 October 2025).
18. Javle, M.; Borad, M.J.; Azad, N.S.; Kurzrock, R.; Abou-Alfa, G.K.; George, B.; Hainsworth, J.; Meric-Bernstam, F.; Swanton, C.; Sweeney, C.J.; et al. Pertuzumab and Trastuzumab for HER2-Positive, Metastatic Biliary Tract Cancer (MyPathway): A Multicentre, Open-Label, Phase 2a, Multiple Basket Study. *Lancet Oncol.* **2021**, *22*, 1290–1300. https://doi.org/10.1016/S1470-2045(21)00336-3.
19. Jazz Pharmaceuticals. NCT02892123—Trial of ZW25 (Zanidatamab) in Patients With Advanced HER2-expressing Cancers. Available online: https://www.clinicaltrials.gov/study/NCT02892123 (accessed on 29 October 2025).
20. Harding, J.J.; Fan, J.; Oh, D.-Y.; Choi, H.J.; Kim, J.W.; Chang, H.-M.; Bao, L.; Sun, H.-C.; Macarulla, T.; Xie, F.; et al. Zanidatamab for HER2-Amplified, Unresectable, Locally Advanced or Metastatic Biliary Tract Cancer (HERIZON-BTC-01): A Multicentre, Single-Arm, Phase 2b Study. *Lancet Oncol.* **2023**, *24*, 772–782. https://doi.org/10.1016/S1470-2045(23)00242-5.
21. Nakamura, Y.; Mizuno, N.; Sunakawa, Y. Tucatinib and Trastuzumab for Previously Treated Human Epidermal Growth Factor Receptor 2-Positive Metastatic Biliary Tract Cancer (SGNTUC-019): A Phase II Basket Study. *J. Clin. Oncol.* **2023**, *41*, 5569–5578. https://doi.org/10.1200/JCO.23.00606.
22. Lee, C.; Chon, H.J.; Cheon, J.; Lee, M.A.; Im, H.-S.; Jang, J.-S.; Kim, M.H.; Park, S.; Kang, B.; Hong, M.; et al. Trastuzumab plus FOLFOX for HER2-Positive Biliary Tract Cancer Refractory to Gemcitabine and Cisplatin: A Multi-Institutional Phase 2 Trial of the Korean Cancer Study Group (KCSG-HB19–14). *Lancet Gastroenterol. Hepatol.* **2023**, *8*, 56–65. https://doi.org/10.1016/S2468-1253(22)00335-1.
23. Ostwal, V.; Mandavkar, S.; Bhargava, P.; Srinivas, S.; Kapoor, A.; Shetty, O.; Kannan, S.; Chaugule, D.; Patil, R.; Parulekar, M.; et al. Trastuzumab Plus Gemcitabine-Cisplatin for Treatment-Naïve Human Epidermal Growth Factor Receptor 2-Positive Biliary Tract Adenocarcinoma: A Multicenter, Open-Label, Phase II Study (TAB). *J. Clin. Oncol.* **2024**, *42*, 800–807. https://doi.org/10.1200/JCO.23.01193.
24. Ohba, A.; Morizane, C.; Ueno, M. Multicenter Phase II Trial of Trastuzumab Deruxtecan for HER2-Positive Unresectable or Recurrent Biliary Tract Cancer: HERB Trial. *Future Oncol.* **2022**, *18*, 2351–2360. https://doi.org/10.2217/fon-2022-0214.
25. Meric-Bernstam, F.; Makker, V.; Oaknin, A.; Oh, D.-Y.; Banerjee, S.; González-Martín, A.; Jung, K.H.; Ługowska, I.; Manso, L.; Manzano, A.; et al. Efficacy and Safety of Trastuzumab Deruxtecan in Patients with HER2-Expressing Solid Tumors: Primary Results from the DESTINY-PanTumor02 Phase II Trial. *J. Clin. Oncol.* **2024**, *42*, 47–58. https://doi.org/10.1200/JCO.23.02005.
26. Harding, J.J.; Piha-Paul, S.A.; Shah, R.H. Antitumour Activity of Neratinib in Patients with HER2-Mutant Advanced Biliary Tract Cancers. *Nat. Commun.* **2023**, *14*, 630. https://doi.org/10.1038/s41467-023-36399-y.
27. Goyal, L.; Zheng, H.; Yurgelun, M.B. A Phase 2 and Biomarker Study of Cabozantinib in Patients with Advanced Cholangiocarcinoma. *Cancer* **2017**, *123*, 1979–1988. https://doi.org/10.1002/cncr.30571.
28. Abou-Alfa, G.K.; Macarulla, T.; Javle, M.M.; Kelley, R.K.; Lubner, S.J.; Adeva, J.; Cleary, J.M.; Catenacci, D.V.; Borad, M.J.; Bridgewater, J.; et al. Ivosidenib in IDH1-Mutant, Chemotherapy-Refractory Cholangiocarcinoma (ClarIDHy): A Multicentre, Randomised, Double-Blind, Placebo-Controlled, Phase 3 Study. *Lancet Oncol.* **2020**, *21*, 796–807. https://doi.org/10.1016/S1470-2045(20)30157-1. Erratum in *Lancet Oncol*. **2024**, *25*, e61. https://doi.org/10.1016/S1470-2045(24)00013-5.
29. Bridgewater, J.; Lopes, A.; Beare, S. A Phase 1b Study of Selumetinib in Combination with Cisplatin and Gemcitabine in Advanced or Metastatic Biliary Tract Cancer: The ABC-04 Study. *BMC Cancer* **2016**, *16*, 153. https://doi.org/10.1186/s12885-016-2174-8.
30. Kim, J.W.; Lee, K.-H.; Kim, J.-W. Enhanced Antitumor Effect of Binimetinib in Combination with Capecitabine for Biliary Tract Cancer Patients with Mutations in the RAS/RAF/MEK/ERK Pathway: Phase Ib Study. *Br. J. Cancer* **2019**, *121*, 332–339. https://doi.org/10.1038/s41416-019-0523-5.
31. Kim, R.D.; McDonough, S.; El-Khoueiry, A.B. Randomised Phase II Trial (SWOG S1310) of Single Agent MEK Inhibitor Trametinib Versus 5-Fluorouracil or Capecitabine in Refractory Advanced Biliary Cancer. *Eur. J. Cancer* **2020**, *130*, 219–227. https://doi.org/10.1016/j.ejca.2020.01.026

32. Ikeda, M.; Ioka, T.; Fukutomi, A. Efficacy and Safety of Trametinib in Japanese Patients with Advanced Biliary Tract Cancers Refractory to Gemcitabine. *Cancer Sci.* **2018**, *109*, 215–224. https://doi.org/10.1111/cas.13438.
33. Lowery, M.A.; Bradley, M.; Chou, J.F. Binimetinib plus Gemcitabine and Cisplatin Phase I/II Trial in Patients with Advanced Biliary Cancers. *Clin. Cancer Res.* **2019**, *25*, 937–945. https://doi.org/10.1158/1078-0432.CCR-18-1927.
34. Doebele, R.C.; Drilon, A.; Paz-Ares, L.; Siena, S.; Shaw, A.T.; Farago, A.F.; Blakely, C.M.; Seto, T.; Cho, B.C.; Tosi, D.; et al. Entrectinib in Patients with Advanced or Metastatic NTRK Fusion-Positive Solid Tumours: Integrated Analysis of Three Phase 1-2 Trials. *Lancet Oncol.* **2020**, *21*, 271–282. https://doi.org/10.1016/S1470-2045(19)30691-6. Erratum in *Lancet Oncol*. **2021**, *22*, e428. https://doi.org/10.1016/S1470-2045(21)00538-6.
35. Bahary, N. NCT02053376—A Phase 2 Trial of Regorafenib as A Single Agent in Advanced and Metastatic Biliary Tract Carcinoma/Cholangiocarcinoma Patients Who Have Failed First-line Chemotherapy. Available online: https://www.clinicaltrials.gov/study/NCT02053376 (accessed on 29 October 2025).
36. Rizell, M.; Andersson, M.; Cahlin, C. Effects of the mTOR inhibitor sirolimus in patients with hepatocellular and cholangiocellular cancer. *Int. J. Clin. Oncol*. **2008**, *13*, 66–70. https://doi.org/10.1007/s10147-007-0733-3.
37. Jung, K.S.; Lee, J.; Park, S.H. Pilot Study of Sirolimus in Patients with PIK3CA Mutant/Amplified Refractory Solid Cancer. *Mol. Clin. Oncol.* **2017**, *7*, 27–31. https://doi.org/10.3892/mco.2017.1272.
38. Verzoni, E.; Pusceddu, S.; Buzzoni, R. Safety profile and treatment response of everolimus in different solid tumors: an observational study. *Future Oncol*. **2014**, *10*, 1611–1617. https://doi.org/10.2217/fon.14.31.
39. Buzzoni, R.; Pusceddu, S.; Bajetta, E. Activity and Safety of RAD001 (Everolimus) in Patients Affected by Biliary Tract Cancer Progressing after Prior Chemotherapy: A Phase II ITMO Study. *Ann. Oncol.* **2014**, *25*, 1597–1603. https://doi.org/10.1093/annonc/mdu175.
40. Lau, D.K.; Tay, R.Y.; Yeung, Y.H. Phase II Study of Everolimus (RAD001) Monotherapy as First-Line Treatment in Advanced Biliary Tract Cancer with Biomarker Exploration: The RADiChol Study. *Br. J. Cancer* **2018**, *118*, 966–971. https://doi.org/10.1038/s41416-018-0021-1.
41. Kim, S.T.; Lee, J.; Park, S.H. Prospective Phase II Trial of Everolimus in PIK3CA Amplification/Mutation and/or PTEN Loss Patients with Advanced Solid Tumors Refractory to Standard Therapy. *BMC Cancer* **2017**, *17*, 211. https://doi.org/10.1186/s12885-017-3196-6.
42. Costello, B.A.; Borad, M.J.; Qi, Y. Phase I Trial of Everolimus, Gemcitabine and Cisplatin in Patients with Solid Tumors. *Invest. New Drugs* **2014**, *32*, 710–716. https://doi.org/10.1007/s10637-014-0096-3.
43. Bendell, J.C.; Rodon, J.; Burris, H.A. Phase I, Dose-Escalation Study of BKM120, an Oral Pan-Class I PI3K Inhibitor, in Patients with Advanced Solid Tumors. *J. Clin. Oncol.* **2012**, *30*, 282–290. https://doi.org/10.1200/JCO.2011.36.1360.
44. McRee, A.J.; Sanoff, H.K.; Carlson, C. A Phase I Trial of mFOLFOX6 Combined with the Oral PI3K Inhibitor BKM120 in Patients with Advanced Refractory Solid Tumors. *Invest. New Drugs* **2015**, *33*, 1225–1231. https://doi.org/10.1007/s10637-015-0298-3.
45. Kim, R.D.; Alberts, S.R.; Peña, C. Phase I Dose-Escalation Study of Copanlisib in Combination with Gemcitabine or Cisplatin plus Gemcitabine in Patients with Advanced Cancer. *Br. J. Cancer* **2018**, *118*, 462–470. https://doi.org/10.1038/bjc.2017.428.
46. Ahn, D.H.; Li, J.; Wei, L. Results of an Abbreviated Phase-II Study with the Akt Inhibitor MK-2206 in Patients with Advanced Biliary Cancer. *Sci. Rep.* **2015**, *5*, 12122.
47. Hollebecque, A.; Houédé, N.; Cohen, E.E.W. A Phase Ib Trial of LY2584702 Tosylate, a p70 S6 Inhibitor, in Combination with Erlotinib or Everolimus in Patients with Solid Tumours. *Eur. J. Cancer* **2014**, *50*, 876–884. https://doi.org/10.1016/j.ejca.2013.12.006.
48. Azaro, A.; Rodon, J.; Calles, A. A First-in-Human Phase I Trial of LY2780301, a Dual p70 S6 Kinase and Akt Inhibitor, in Patients with Advanced or Metastatic Cancer. *Invest. New Drugs* **2015**, *33*, 710–719. https://doi.org/10.1007/s10637-015-0241-7.

49. Angevin, E.; Cassier, P.A.; Italiano, A. Safety, Tolerability and Antitumour Activity of LY2780301 (p70S6K/AKT Inhibitor) in Combination with Gemcitabine in Molecularly Selected Patients with Advanced or Metastatic Cancer: A Phase IB Dose Escalation Study. *Eur. J. Cancer* **2017**, *83*, 194–202. https://doi.org/10.1016/j.ejca.2017.06.036.
50. Tan, E.S.; Cao, B.; Kim, J. Phase 2 Study of Copanlisib in Combination with Gemcitabine and Cisplatin in Advanced Biliary Tract Cancers. *Cancer* **2021**, *127*, 1293–1300. https://doi.org/10.1002/cncr.33364.
51. Javle, M.M.; Oh, D.-Y.; Ikeda, M. Varlitinib plus Capecitabine in Second-Line Advanced Biliary Tract Cancer: A Randomized, Phase II Study (TreeTopp). ESMO Open **2022**, *7*, 100314. https://doi.org/10.1016/j.esmoop.2021.100314.
